# Optical coherence elastography in ophthalmology

**DOI:** 10.1117/1.JBO.22.12.121720

**Published:** 2017-12-23

**Authors:** Mitchell A. Kirby, Ivan Pelivanov, Shaozhen Song, Łukasz Ambrozinski, Soon Joon Yoon, Liang Gao, David Li, Tueng T. Shen, Ruikang K. Wang, Matthew O’Donnell

**Affiliations:** a University of Washington, Department of Bioengineering, Seattle, Washington, United States; b Akademia Górniczo-Hutnicza University of Science and Technology, Krakow, Poland; c University of Washington, Department of Chemical Engineering, Seattle, Washington, United States; d University of Washington, Department of Ophthalmology, Seattle, Washington, United States

**Keywords:** optical coherence tomography, optical coherence elastography, acoustic radiation force, air-coupled ultrasound, phase-sensitive optical coherence tomography, tissue elasticity, ocular biomechanics, speckle tracking, mechanical wave imaging

## Abstract

Optical coherence elastography (OCE) can provide clinically valuable information based on local measurements of tissue stiffness. Improved light sources and scanning methods in optical coherence tomography (OCT) have led to rapid growth in systems for high-resolution, quantitative elastography using imaged displacements and strains within soft tissue to infer local mechanical properties. We describe in some detail the physical processes underlying tissue mechanical response based on static and dynamic displacement methods. Namely, the assumptions commonly used to interpret displacement and strain measurements in terms of tissue elasticity for static OCE and propagating wave modes in dynamic OCE are discussed with the ultimate focus on OCT system design for ophthalmic applications. Practical OCT motion-tracking methods used to map tissue elasticity are also presented to fully describe technical developments in OCE, particularly noting those focused on the anterior segment of the eye. Clinical issues and future directions are discussed in the hope that OCE techniques will rapidly move forward to translational studies and clinical applications.

## 1 Introduction

**Fig. 1:**
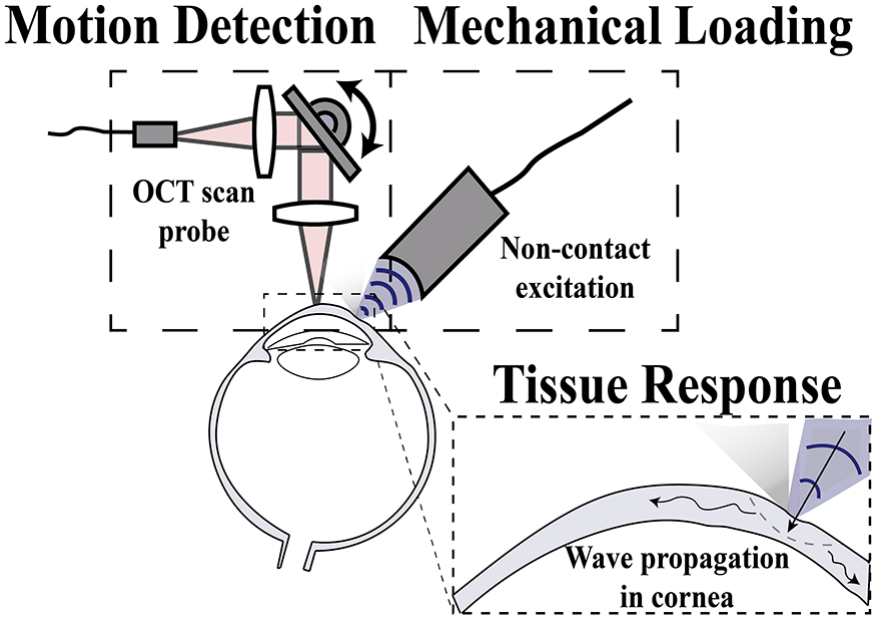
Schematic demonstrating the main components of OCE.

Optical coherence tomography (OCT) has outstanding spatial resolution, noncontact operation, and sufficient penetration depth in ocular tissues, making it a nearly perfect match for ophthalmologic applications. In addition to micron-level spatial resolution, OCT can detect tissue motion with exquisite sensitivity, making it feasible for *in vivo* sensing of micro- and nanoscale displacement within a sample. Because of this, OCT can be used in specific configurations to probe biomechanical properties, a technique referred to as optical coherence elastography (OCE).^1^ In theory, OCT generates a structural image based on light scattering determined by minute changes in the refractive index of different tissue and cell types, while OCE utilizes local tissue motion as a function of an applied stress to infer tissue stiffness (i.e., elasticity). An OCE system may be described in terms of the steps required to probe tissue elasticity: (1) mechanical loading, (2) tissue response, and (3) motion detection, as shown in Fig. 1.

Biomechanical testing can be used in ophthalmology to probe structural characteristics in both diseased and healthy tissues that are difficult to contrast using classical OCT methods. For example, light backscattering within multiple tissue sections may be the same but exhibit different elastic modulus. As disease can affect the structural organization and function of human cells, collagen fibers, and extracellular matrix, changes in local elastic moduli may be used to diagnose and help manage treatment of diseased tissue within the cornea, sclera, lens, and retina.^2^^–^^5^ Thus, the Young’s modulus, E, is of particular interest in ophthalmology due to large differences in biomechanical properties of ocular tissues reported in various pathological states (Table 1). However, reliable measurements of ocular mechanical properties have historically been possible only with *ex vivo* tissue and have not yet directly impacted the clinic.^3^ In human tissue, reported values of E are often for both *in vivo* and *in vitro* tissues, and at length scales determined by the method of evaluation, which can provide potentially ambiguous quantitation depending on the experimental conditions. Additionally, many reported *in vivo* values have not been validated since the techniques used to measure them are so new. As the relative differences between healthy and diseased tissues are large enough to potentially be clinically relevant, there is a clear need to validate and standardize *in vivo* methods capable of robust and reliable measurement of modulus.

**Table 1:** Reported values of Young’s moduli for ophthalmic tissue types in various physiologic states, at physiologically relevant pressures.

Tissue type	Measurement condition	Disease state	Young’s modulus (Pa)
Cornea (human)	Tensile testing,^6^	Normal	$0.29\times 10^{6}$ to $1.3\times 10^{6}$
	Orssengo–Pye algorithm,^7^	Collagen crosslinking	$5.9\times 10^{6}$
			$2.5\times 10^{4}$ to $40\times 10^{4}$
	Inflation testing^8^	Middle age (50 to 64)	
			$20\times 10^{4}$ to $58\times 10^{4}$
		Old age (65 to 05)	
Cornea (porcine)	OCE (air-puff)^9^^–^^11^	Normal	5.9 to $52\times 10^{3}$
		Collagen crosslinking	43.9 to $110\;\times \;10^{3}$
Anterior lens capsule (human)	Atomic force microscopy^12^	Normal	$0.485\times 10^{6}$
		Cataract	$0.416\times 10^{6}$
	Tensile testing^13^	Young age	$27.4\times 10^{6}$
		Old age	$3.3\times 10^{6}$
Lens nucleus (human)	Microbubble-based acoustic radiation force^14^	Middle age (40- to 50-years old)	0.19 to $5.2\times 10^{3}$
			3 to $10.6\times 10^{3}$
	Spin test^15^^,^^16^	Old age (63- to 70-years old)	
Retinal tissue (bovine)	Mathematical model^17^	Healthy	$2.6\times 10^{4}$
	OCE (ARF)^18^	Healthy	$6.2\times 10^{3}$
Retinal tissue (porcine)			
Sclera	Tensile testing^19^	Healthy	$2.6\times 10^{6}$
	Biaxial tensile testing^20^		$14\times 10^{6}$

Elastography began with ultrasound and MRI as the means to image relatively large tissue motion related to local mechanical properties.^21^^–^^29^ However, MRI has inherently low spatial resolution with long imaging times often not appropriate for clinical applications in the eye. More recently, an ultrasonic technique based on supersonic shear wave imaging (SSI) was introduced for corneal stiffness estimation, where SSI was performed on both *ex vivo* and *in vivo* porcine cornea, producing two- (2-D) and three-dimensional (3-D) maps of cornea stiffness.^30^ However, SSI requires direct contact for acoustic coupling with the cornea, a feature that inherently causes patient discomfort. It also has relatively low spatial resolution that remains largely limited by the host imaging modality of ultrasonography.^31^

OCT, on the other hand, is a near-perfect candidate for imaging microscale displacements related to mechanical properties within relatively transparent layers of the cornea and lens. Recent advances in high-speed noncontact excitation and detection methods have made high-resolution OCE not only possible but also plausible for clinical translation. To detect stiffness-related motion within a tissue substrate, OCT has been utilized in a number of configurations, with appropriate scanning protocols and processing techniques based on speckle tracking and phase-sensitive OCT (PhS-OCT), a subject discussed in Secs. 3 and 4 of this review.

**Fig. 2:**
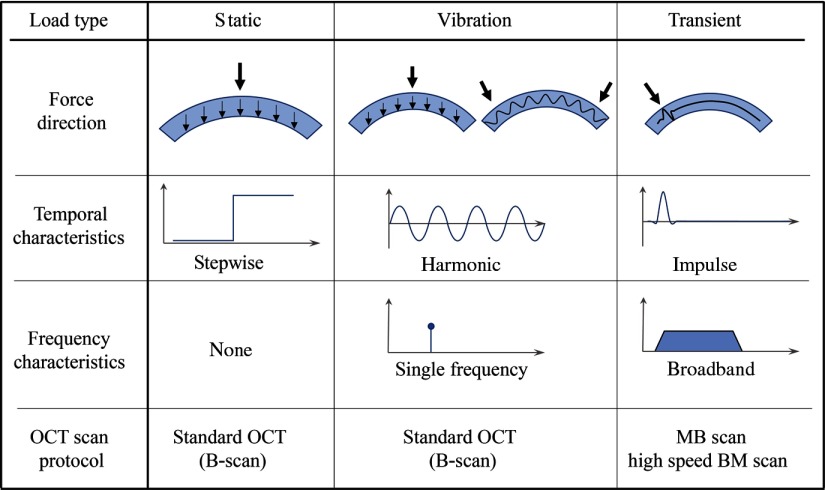
Typical loading schemes in OCE.

Multiple OCT motion detection approaches have been used in OCE, all of which stem from a user-defined constitutive equation for Young’s (or shear) modulus, derived from a mechanical model. The appropriate OCT configuration, and thus the applicable constitutive equation, depends on the method used to induce local tissue displacements. Typical approaches can be characterized as static, or quasistatic compression,^1^^,^^32^^–^^36^ vibration,^37^^–^^39^ and transient excitation (wave propagation),^40^^–^^47^ each of which is discussed in Sec. 2. The specific method used to estimate tissue elasticity from localized motion must be considered at great length when choosing an excitation method. To this point, multiple methods have been used to mechanically load ocular tissue, including a curved plate,^48^ wire-tip,^46^^,^^49^ air-puff,^50^^–^^53^ optical excitation,^54^ acoustic radiation force (ARF),^18^^,^^55^^,^^56^ and air-coupled ARF [or acoustic microtapping (AμT)].^57^^,^^58^ For visual reference, a summary of the more prevalent characteristics of common excitation and detection methods used in OCE are presented in Fig. 2.

Any coupling medium is a practical challenge for translational ophthalmic research. A truly noncontact system developed to both induce and measure small displacements within the eye will likely have the fastest path to clinical adoption. Because of this, transient excitation methods using a mechanical wave disturbance paired with high-speed OCT appear to be the most translatable in probing ocular biomechanics. Recently, methods based on transient excitation (namely an air-puff OCE system^59^ and a contact-based loading method utilizing anesthetic drops^60^) demonstrated the ability to evaluate the mechanical properties of human cornea in a clinical setting as an initial step toward translatable OCE systems. However, there remain numerous practical and theoretical issues regarding wave excitation and behavior that must be addressed to deduce a meaningful, quantitative measure of tissue stiffness, a topic discussed in Sec. 2 of this review.

As OCT technology continues to advance, we expect OCE’s impact on ophthalmology to grow. Over the past 25 years, OCT has revolutionized ophthalmology by enabling noninvasive evaluation of the posterior as well as anterior segments of the human eye. Much like how OCT revolutionized retinal diagnosis and treatment monitoring, we expect OCE to follow suit as a valuable clinical tool for the anterior segment. Recent advances in OCT light sources, scanning protocols, methods to detect micron- and nanoscale motion, and sources of noncontact excitation have led to near real-time optical elastography of ocular tissues for practical clinical application.

A properly designed OCE system can complement other modalities for clinical applications in the anterior segment of the eye. Differences in elasticity can help both identify tissue types related to disease and monitor therapeutic interventions, similar to other medical imaging systems using multiple contrast mechanisms. That is, elasticity images at the microscale can identify changes in specific tissue types within the eye. Unlike most imaging systems, however, OCE has the potential to quantitatively map the elastic modulus with sub-mm to micron resolution, providing the information needed to drive biomechanical models predicting shape changes in the primary focusing apparatus of the eye. If this can be demonstrated in the clinic, then OCE can help guide and optimize therapeutic interventions on a personalized basis with a precision beyond the current state-of-the-art. This prospect is an exciting goal for translational research in OCE.

In this review, we describe the fundamental principles of OCE as applied in ophthalmology, noting limitations that must be considered in developing the technology for practical applications primarily in the anterior segment. We describe the OCT methods used in system design to detect tissue displacement. Coupling theory with system design, we then describe multiple research areas that have already used OCE to probe biomechanics in clinically relevant arrangements and touch on unrealized areas of research application. The goal is to address some recent advances in OCE specific to ophthalmology while providing a framework for future technological development. We refer the reader to a number of reviews that touch on a wider application space.^43^^–^^45^^,^^61^

## 2 OCE Displacement Methods

Elasticity imaging requires a physical stress to deform (displace) tissue. Resulting displacements are measured with OCT to compute strains, detect vibrations, or track the propagation of a mechanical wave. The stress–strain response of tissue, local vibration behavior, or mechanical wave content, can each be mapped spatially to solve for metrics such as the Young’s (or shear) modulus. Applicable constitutive equations and their corresponding excitation methods are defined within this section.

### 2.1 Static Displacement Methods

**Fig. 3:**
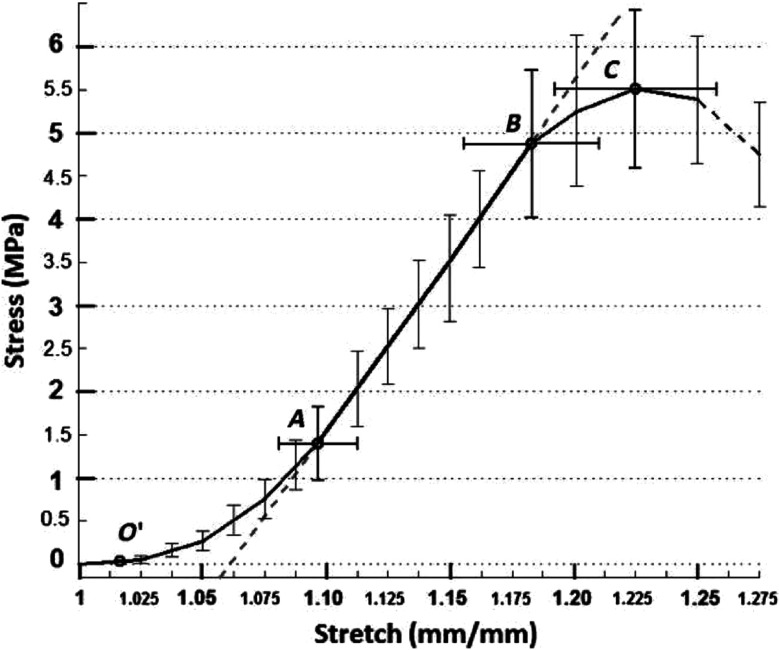
Mean stress–strain curves measured in porcine cornea under tensile loading. The region between $O^{\prime }$ and A is described as the region of crimped collagen fibers; A to B is the ‘linear region’ of aligned collagen fibers; and B to C is nonlinear as damage begins. (Figure reproduced from Ref. 62.)

Tissue elastic properties can be described by the instantaneous deformation response following an applied mechanical stress. The classic approach to quantify elastic modulus uses tensile testing to induce a known stress while measuring strain. An example of a typical stress–strain curve of the cornea is presented in Fig. 3. Under increasing strain, the collagen fibers defining tissue structure are typically crimped under low strain, but expand until aligned. The curvature in the stress–strain response is thus nonlinear until fibers are aligned, where the tissue begins to demonstrate near-linear elastic deformation until the point of material failure.^62^ Linear elastic materials have a constant elastic modulus (equal to the slope of the stress–strain curve as an incrementally increasing stress is applied), while nonlinear elastic materials have an elastic modulus that changes under different strains (i.e., the slope of the stress–strain curve changes between incrementally different applied stresses). For *in vivo* applications, the stress–strain curve may differ from that in Fig. 3 due to the preload delivered by the intraocular pressure (IOP). In many cases, such preload naturally stretches corneal collagen fibers to the point of linear deformation.

As ocular tissue is typically measured under preload over only a few percent strain, the sample is often assumed to be within the linear regime, where stress has approximately a linear effect on strain. Assuming a linear isotropic material, the elastic modulus is easily related to the shear modulus and tensile stress is equal to compressive stress. Following the small-deformation assumption, applied static axial force results in axial displacements that can be used to calculate the Young’s modulus based on the strain detected using OCT. For an infinite, homogeneous media under a uniform mechanical load, the stress is assumed to be equally distributed and dispersed throughout the sample. Under these conditions, the stress can be defined as the force over a given area

$$\sigma =\frac{F}{A}.$$ (1)

In reality, force applied at a specific location results in a stress distribution throughout the tissue in 3-D, requiring second- and fourth-order tensor terms to fully determine the elastic properties of a material.^63^ Deformations can be computed using Newton’s second law applied to the elastic constitutive relations subject to the mechanical boundary conditions applied to the object under study. For an isotropic, homogeneous elastic material, the full constitutive relations between stress, σ, and strain, ϵ, can be expressed in terms of two independent moduli, the Lame constants (λ,μ), as

$$\sigma_{ij}=\lambda \epsilon_{ij}\delta_{ij}+2\mu \epsilon_{ij},$$ (2)

having the corresponding matrix notation

$$[\begin{array}{c} \sigma_{11}\\ \sigma_{22}\\ \sigma_{33}\\ \sigma_{23}\\ \sigma_{13}\\ \sigma_{12} \end{array}]=[\begin{array}{cccccc} \lambda +2\mu & \lambda & \lambda & & & \\ \lambda & \lambda +2\mu & \lambda & & 0 & \\ \lambda & \lambda & \lambda +2\mu & & & \\ & & & \mu & & \\ & 0 & & & \mu & \\ & & & & & \mu \end{array}][\begin{array}{c} \epsilon_{11}\\ \epsilon_{22}\\ \epsilon_{33}\\ 2\epsilon_{23}\\ 2\epsilon_{13}\\ 2\epsilon_{12} \end{array}],$$ (3)

where λ is the first Lame constant, μ is the shear modulus, i and j represent the Cartesian coordinates, and $\delta_{ij}$ is the Kronecker delta function. The symmetric strain tensor ϵ (second rank) is defined as the differential change in the components of the displacement vector ($\overset{\to }{U}$) with respect to space coordinates $\left(x_{i},x_{j}\right)$, where the individual components are

$$\epsilon_{ij}=\frac{1}{2}[\frac{dU_{i}}{dx_{j}}+\frac{dU_{j}}{dx_{i}}].$$ (4)

For soft tissues, Eqs. (2) and (3) can be simplified. Indeed, most soft tissues, including those considered in ophthalmology, can be considered incompressible, i.e., their volume is conserved under load [i.e., Tr(ϵ)≡0].

In practice, soft tissues are not completely incompressible, but their shear modulus, μ, is typically several orders of magnitude smaller than λ. Thus, to avoid possible confusion, we will use the terminology “nearly” incompressible media to describe soft tissue. Calculating Tr(σ) from Eq. (3) and taking into account^64^

Tr(σ)=−3p, (5)

and both μ≪λ and Tr(ϵ)→0, we obtain

$$p≅-\lambda (\epsilon_{11}+\epsilon_{22}+\epsilon_{33}).$$ (6)

Here, p is the internal hydrostatic pressure representing the stable product of a very large modulus (λ) with a vanishing trace of the strain matrix. It can vary both in space and time as determined by the loading function and the boundary conditions. Therefore, constitutive relations (2) and (3) for a nearly incompressible soft tissue reduce to

$$\sigma_{ij}=-p\delta_{ij}+2\mu \epsilon_{ij}.$$ (7)

The pressure term, p, points to the relationship between IOP and the stress/strain relations determined by the modulus. We note in general that the modulus itself can also be a function of the preload over large pressure ranges based on the nonlinear stress–strain relation over this range.

Although the pressure must be taken into account for general 3-D loads, the stress–strain relation simplifies for the case of a unidirectional (1-D) load, i.e.,

$$\sigma =[\begin{array}{ccc} 0 & & 0\\ & 0 & \\ 0 & & \sigma_{33} \end{array}].$$ (8)

The stress–strain relations simplify to

$$\sigma_{33}=-p+2\mu \epsilon_{33}\;0=-p+2\mu \epsilon_{11}\;0=-p+2\mu \epsilon_{22}.$$ (9)

Taking into account that Tr(ϵ)≡0, the pressure term can be eliminate and the stress–strain relation reduces to

$$\sigma_{33}=3\mu \epsilon_{33}.$$ (10)

Equation (10) finally defines the linear relationship between applied 1-D stress and the axial strain along the same direction, where the proportionality constant is the elastic modulus. Thus, for nearly incompressible soft tissue

σ=3μϵ=Eϵ, (11)

where E is the Young modulus (i.e., elasticity). Equation (11) indicates that any statically deformed, isotropic soft tissue can be completely characterized by the spatial distribution of a single material parameter, either shear (μ) or Young’s (E) modulus. The Young’s modulus, E, is a relatively intuitive parameter, as a material that requires a higher stress to induce a fixed deformation would physically feel stiff to the touch compared to another material requiring a smaller stress for the same deformation.

To create a final elasticity image using a quantitative modulus value, stress and strain must both be defined at each location within tissue. The 1-D localized stress may be related to material strain through the spatial derivative of the displacement over a region in the Z direction of the applied load, and easily measured and mapped spatially using OCT (see Secs. 3 and 4). Such mapping based on local motion detected under 1-D axial loading has been demonstrated on human cornea utilizing a modified goniometer.^48^

As the end goal of OCE is to accurately determine the ratio between stress and strain at a local region, the local stress distribution must be well defined. One source of complication is that for a given stress applied to the surface, the internal stress distribution can be complicated and difficult to quantify. For example, in a homogeneous medium, higher stresses are distributed closer to the applied load. Because the stress distribution is often unknown, the measured strain cannot be simply inverted to quantify mechanical properties. A reported attempt to accurately quantify the uneven (and unknown) stress within tissue utilized a compliant stress sensor to map the force (per unit area) distribution near the surface of the sample;^33^ however, the depth-resolved stress distribution remains unknown.

Another source of ambiguity when measuring ocular tissue stiffness is the poorly defined preload within tissue determined by the host IOP. Patient-specific variations in host IOP may place the tissue under a different preload, producing different modulus values under the same applied force. Also, the pressure term in the constitutive relation of Eq. (7) cannot be simply removed as in the 1-D case for general 3-D loading. Thus, the pressure term must be considered in addition to the strain values to calculate a robust quantitative modulus value for ocular tissue.

While static methods have been used to probe tissue stiffness, such as in *ex vivo* tumor detection,^32^^,^^65^^,^^66^ they have not been as widely developed in ophthalmology due, in part, to the contact needed to apply an even force across the tissue surface. In general, static methods can produce images providing contrast related to tissue elasticity, but struggle to provide robust and reliable quantitative maps of the Young’s modulus in ocular tissues. Additionally, static-methods are often used to probe elastic properties when, in reality, the physical response in human tissue depends on the rate at which the tissue is loaded. Thus, viscoelastic analysis is possibly based on the time-dependent rate of deformation.^67^^,^^68^ In general, the elastic response can be thought of as the static tissue response to a force while the viscoelastic response describes the dynamic properties of the material and is often described by the relaxation time, or hysteresis, and can potentially provide additional information regarding the state of the tissue under investigation.^2^^,^^3^ Time-dependent, or cyclical, loading may be used to determine both the elastic and viscous components of tissue, as discussed in the next section.

### 2.2 Steady-State Harmonic Methods

The mechanical response of human tissue under load contains a viscous component producing a time-dependent strain response to an applied stress. This time-dependent response can be used to probe both elastic and viscous components by cyclically loading and unloading the region under investigation at a controlled rate. Both components provide information about tissue type and can be used to generate images related to the viscoelastic properties of tissue.

**Fig. 4:**
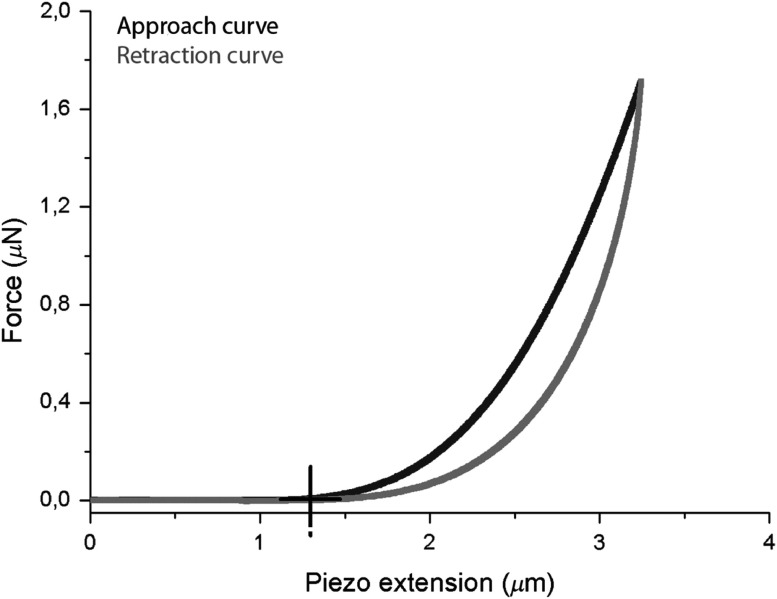
Differences in the load and unload pattern define hysteresis. The time for a deformed tissue to return to its original position is the relaxation time. (Figure reproduced from Ref. 69).

As viscoelastic materials exhibit strain rate-dependent responses, different frequency loading schemes can be used to probe the time-dependent deformation based on either local hysteresis or rate of recovery.^37^^,^^38^ The recovery rate can be defined as the time it takes for a deformation to return to its original position. In other words, viscous materials may exhibit a slower relaxation rate compared to the induced deformation rate in certain cases. In the cornea for example, fiber rearrangement under load often produces corneal relaxation that differs from the induced deformation response. Such rearrangement often produces a measurably different strain (hysteresis) and relaxation rate in the unloading cycle compared to the loading cycle, as demonstrated on corneal stroma tissue in Fig. 4. The difference between loading and unloading stress–strain curves represents energy lost to viscous deformation and can be used to infer viscosity at low mechanical frequencies. Because of viscous damping, different loading and unloading rates result in unique tissue responses that can be described only with both elastic and viscous terms.

When a force is applied to the sample surface, energy is transferred and distributes spatially, similar to a wave disturbance: a phenomenon that has been well studied in physical acoustics. We provide a more detailed description of wave behavior in the next section, but introduce the concept here to show how time-dependent loading can induce tissue vibrations. As a sample is subject to cyclical loading and unloading, energy travels throughout the medium, loading and unloading local tissue at a rate set by the driving frequency. A number of recent techniques have been developed that exploit the time-dependent response to an applied force, including low-frequency excitation,^38^ swept frequency excitation,^37^ acoustic vibrography,^70^^,^^71^ and multiple source excitation (crawling wave),^72^ but have had limited use for ocular tissue. Viscoelastic properties have also been probed using magnetic nanoparticles embedded in tissue that vibrate when subject to electromagnetic forces.^73^ However, magnetomotive methods use particle inclusions, which may be difficult to incorporate in ocular tissue such as the cornea.

In cyclic methods, low-frequency (sub-kHz) loads are often used. As the axial force is cycled at a single frequency, a loss, or storage, modulus may be used to describe the phase difference between applied stress and strain response in a viscoelastic material. That is, the strain response typically lags the applied stress. The storage (elastic) component of the Young’s modulus, $E_{1}$, and the loss (viscous) component, $E_{2}$, can thus be defined as^74^

$$E_{1}=\frac{\sigma }{\epsilon }*\cos\;\delta ,$$ (12)

$$E_{2}=\frac{\sigma }{\epsilon }*\sin\;\delta ,$$ (13)

where δ defines the phase of the tissue response at a specific mechanical frequency. The dynamic complex modulus (see Sec. 2.3.5 for details) can then be calculated as

$$\overset{˜}{E}=E_{1}+iE_{2},$$ (14)

where the modulus is an explicit function of frequency. As the storage and loss moduli depend on the out of phase response, testing at different frequencies is often required to accurately measure the dynamic response.

One corollary of Eqs. (12) and (13) is that a resonance occurs within tissue when storage and viscous components are loaded in-phase, resulting in relatively larger strains. The in-phase responses, often defined as the natural frequencies of mechanical resonance, are related to material properties and may be detected by sweeping the induced vibration frequencies.^71^ Assuming a perfectly homogeneous medium with known geometrical constraints, the resonant frequency and Young’s modulus are simply related.^39^ Under these assumptions, further derivation of the natural frequency equation can give the damped natural frequency and can also be used to infer elastic modulus.^75^ The resonance effect may be further exploited using multiple excitation sources to vibrate a sample at slightly different frequencies centered around the natural frequency. The superposition of multiple vibration modes between sources produces a slower “crawling wave”^72^ that may be detected with OCT and used to infer elastic moduli. Similarly, by vibrating the tissue at the resonance frequency in multiple locations, the displacement profile in time may be used to detect a “standing” mechanical wave to infer elastic modulus.^42^ The displacement amplitude among loads has also been used to quantify recovery rate and hysteresis.^76^

A problem with harmonic methods is that the resonance frequency greatly depends upon material geometry. As energy is transferred into the sample, the vibrational energy reflects off internal surfaces, resulting in a resonance that may be dominated by the distance between reverberations and not necessarily by the intrinsic mechanical properties of that tissue.^77^ Thus, the geometry must be taken into account explicitly to calculate the elastic modulus based on natural frequency. The relationship between actual strain and strain rate, akin to the relation between displacement and velocity (as is measured with OCT, see Sec. 3), is also unclear. Additionally, steady-state methods use only a few frequencies (typically at very low frequencies) to infer elastic and viscous modulus.

Because of ambiguities in the stress–strain relationships (due to preload and geometric constraints, for example), the unique time-dependent response of viscoelastic materials requires that samples be probed at different strain rates to fully quantify $E_{1}$ and $E_{2}$ in Eq. (14). Low frequency, or single frequency, methods are thus not robust enough to accurately distinguish complex moduli in many different types of tissue. For robust assessment of the complex modulus in an unknown sample, a range of frequencies must be used to analyze tissue response, severely limiting system speed and overall clinical applicability of steady-state methods. Analysis based on broadband vibrations is generally better suited to tissue biomechanical analysis, as described in the next section. Nevertheless, harmonic methods may provide images with contrast directly related to the viscoelastic properties of tissue.

### 2.3 Propagating Mechanical Waves

When a load induces a temporally short axial displacement at a tissue surface, a mechanical wave is launched within the sample. An isotropic, elastic solid material can support two types of bulk waves, i.e., longitudinal, in which the particles’ oscillations are in the direction of wave propagation, and shear, in which motion is perpendicular to the propagation direction. The resulting wave behavior (i.e., speed, dispersion, attenuation, etc.) depends on the mechanical properties and can thus be used to probe local elasticity. One benefit is that the internal stress distribution is not needed to reconstruct the modulus. The tradeoff, however, is that the relationship between wave behavior and elastic modulus is often complicated by conditions such as sample geometry, boundary conditions, and excitation parameters. For example, human tissue is often layered, resulting in a wave in bounded media containing multiple modes guided along the layer interface. The speed of the resulting guided waves depends on mechanical properties of the adjacent media, boundary conditions, and geometrical features of the interface in addition to the host modulus.^78^^,^^79^ Understanding mechanical wave motion is fundamental for successful OCE implementation; therefore, a short background related to various modes of elastic wave propagation is presented below.

#### 2.3.1 Bulk waves

First, consider a semi-infinite, homogeneous, nearly incompressible elastic material. This fundamental case can be used to describe the basic principles of wave excitation and propagation when the thickness of tissue is much larger than the wavelength of propagating mechanical waves. In other words, we consider bulk wave modes independent of boundary conditions. Although this is rarely the case in ocular tissue, we use this simple case as the starting point to discuss shear wave propagation.

Applying Newton’s second law to the constitutive relations for an isotropic material yields the equation of motion

$$\rho \frac{\partial^{2}\overset{\to }{U}}{\partial t^{2}}=\mu \Delta \overset{\to }{U}+(\lambda +2\mu )\mathrm{grad}\;\mathrm{div}\;\overset{\to }{U},$$ (15)

where ρ is the mass density, (λ,μ) are the Lame constants (defined above), t is the time, and $\overset{\to }{U}$ is the displacement vector of the propagating wave, which can be represented as

$$\overset{\to }{U}={\overset{\to }{U}}_{l}+{\overset{\to }{U}}_{s}.$$ (16)

The longitudinal displacement (${\overset{\to }{U}}_{l}=\mathrm{grad}\;\phi$) and shear displacement (${\overset{\to }{U}}_{s}=\mathrm{rot}\;\psi$) are defined in terms of a scalar potential ϕ and vector potential ψ. Using these definitions, longitudinal (i.e., compressional) and shear waves satisfy independent equations of motion

$$\rho \frac{\partial^{2}{\overset{\to }{U}}_{l}}{\partial t^{2}}-(\lambda +2\mu )\Delta {\overset{\to }{U}}_{l}=0\;\rho \frac{\partial^{2}{\overset{\to }{U}}_{s}}{\partial t^{2}}-\mu \Delta {\overset{\to }{U}}_{s}=0.$$ (17)

The term (λ+2μ) is defined as the p-wave modulus, B, of the material and is related to the wave speed for longitudinal (compressional) waves

$$C_{l}=\sqrt{B/\rho }.$$ (18)

Similarly, shear waves propagate at wave speed determined solely by the shear elastic modulus

$$C_{s}=\sqrt{\mu /\rho }.$$ (19)

For nearly incompressible materials, the p-wave modulus is orders of magnitude greater than the shear modulus. Thus, compressional waves travel at speeds much faster than shear waves and can be ignored over the time scales considered in OCE (i.e., compressional waves propagate far into the medium by the time measurable shear waves begin to propagate). In the remainder of this section, we focus primarily on modes propagating near the shear wave speed, as they travel at rates that can be captured with specially designed OCT systems. Note that the bulk shear wave speed is simply related to the shear (Young’s) modulus and, therefore, it can lead directly to a quantitative estimate of tissue elasticity. Unfortunately, in most ocular tissues, pure bulk shear waves cannot be identified over the range of frequencies used in OCE since boundary conditions can significantly affect wave speeds.

#### 2.3.2 Rayleigh waves

**Fig. 5:**
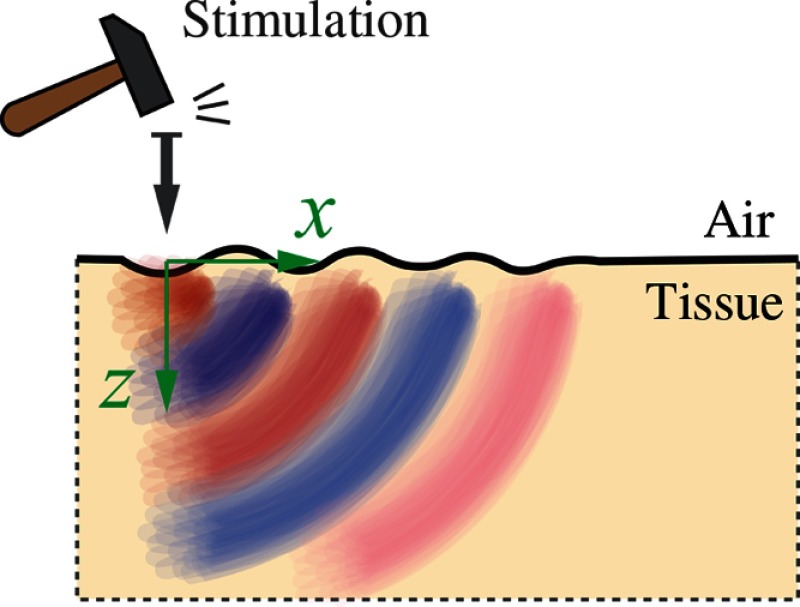
Rayleigh mechanical waves propagate along the air/tissue interface.

Mechanical excitation at a free (i.e., air-material) boundary can produce mechanical disturbances propagating along the medium surface (see Fig. 5) in addition to bulk waves traveling at longitudinal and shear speeds within the medium.

A mechanical disturbance along the medium surface is called a Rayleigh wave and must satisfy the boundary condition that longitudinal and shear stress components at the surface (based on the displacement component perpendicular to the surface) must be zero. For the 2-D (x-z plane), that is,

$${\sigma_{xz}^{\text{tissue}}|}_{z=0}=0\;{\sigma_{zz}^{\text{tissue}}|}_{z=0}=0,$$ (20)

at the tissue surface. Given this boundary condition and the equations of motion, the Rayleigh wave number obeys the polynomial equation

$$\eta^{6}-8\eta^{4}+8(3-2\xi^{2})\eta^{2}-16(1-\xi^{2})=0,$$ (21)

where $\eta =C_{R}/C_{s}$ and $\xi =C_{s}/C_{l}$. Taking into account that ξ≪1 for nearly incompressible materials such as soft tissue (including all ocular tissues), this equation reduces to

$$\eta^{6}-8\eta^{4}+24\eta^{2}-16=0,$$ (22)

with primary root

$$\eta =C_{R}/C_{s}≅0.955$$ (23)

corresponding to the Rayleigh wave ($C_{R}$ is the Rayleigh wave speed), which, like the pure shear wave, depends only on the shear modulus, μ.^78^ Thus, tracking the propagation of either shear or Rayleigh waves to locally estimate wave speed can be used to infer the shear modulus, μ, and, therefore, tissue elasticity (E=3μ).

#### 2.3.3 Scholte waves

**Fig. 6:**
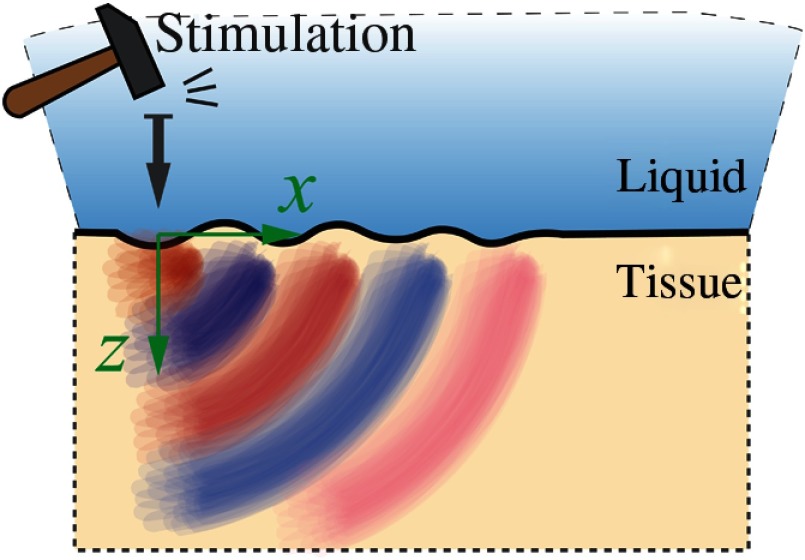
Scholte mechanical waves propagate along the liquid/tissue interface.

Shear and Rayleigh waves are sufficient to describe propagation in the region of a free boundary. However, only the cornea has a true free-mechanical boundary (air–tissue). Other ocular tissues can be better described with a liquid–tissue boundary. For example, consider the intraocular lens. From a mechanical viewpoint, it can be considered a semi-infinite nearly incompressible elastic medium loaded with liquid from the top (see Fig. 6).

Shear waves do not propagate in the liquid, but the load changes the boundary conditions for a propagating interface wave. In this case, the normal stress component is equal to the pressure in the liquid, and the normal displacement components in the tissue must be equal at the interfaces; the tangential component of stress is again taken as zero since fluids do not resist shear deformation

$${\sigma_{xz}^{\text{tissue}}|}_{z=0}=0\;{\sigma_{zz}^{\mathrm{liq}}|}_{z=0}={\sigma_{zz}^{\text{tissue}}|}_{z=0}\;{U_{z}^{\mathrm{liq}}|}_{z=0}={U_{z}^{\text{tissue}}|}_{z=0},$$ (24)

where the relationship μ=0 is taken into account for the liquid.^78^

A liquid–solid interface wave is often called a Scholte wave, obeying the polynomial equation for $\zeta =C_{\mathrm{Sch}}/C_{s}$

$$\zeta^{6}{\left(1+\beta \right)}^{2}-8\zeta^{4}(1+\beta )+24\zeta^{2}+8\zeta^{2}\beta -16=0,$$ (25)

where $C_{\mathrm{Sch}}$ is the speed of the Scholte wave, and (using the lens as an example) $\beta =\rho_{\mathrm{liq}}/\rho_{\text{lens}}$. Noting that the densities of soft tissue (lens and liquid in this case) are approximately the same, i.e., β≅1, the numerical solution of the above equation gives the root

$$\zeta =C_{\mathrm{Sch}}/C_{s}≅0.846.$$ (26)

In other words, liquid loading slows the Rayleigh wave, converting it to a Scholte wave.^78^ Again, the speed of this mode can also be characterized with a single elastic coefficient. Note also that the liquid load allows a leaky wave solution. However, the similarities in bulk properties of liquid and intraocular lens, and the high attenuation of leaky waves, make them very inefficient for tracking.

Rayleigh and Scholte waves have elliptical polarizations because both the $U_{x}$ and $U_{z}$ components of the waves are generally nonzero and π/2 phase-shifted.^79^ However, the vertical, $U_{z}$, component of the displacement is typically utilized as it is more easily detected by PhS-OCT (See Sec. 3.2) in an OCE system with vertically aligned OCT probes.

For the bulk and surface modes considered so far, wave propagation is purely elastic and there is no dispersion in the wave speed (i.e., frequency independent). In the next two sections, we consider dispersive propagation induced both by geometry (bounded waves) and material properties (viscosity). To obtain quantitative estimates of the elastic modulus at very low frequencies approaching zero frequency (i.e., DC limit representing tissue elasticity), dispersion must be taken into account using wideband measurements of mechanical wave propagation. In Sec. 2.3.4, we note the theoretical complexity associated with guided wave propagation in the cornea, and describe how wideband measurements of wave propagation can potentially be used to extract tissue elasticity for this complicated case. In Sec. 2.3.5, we consider material-dependent dispersion due to viscosity. Experimental studies paralleling this analysis are presented briefly in Secs. 4 and 5.

#### 2.3.4 Guided waves

**Fig. 7:**
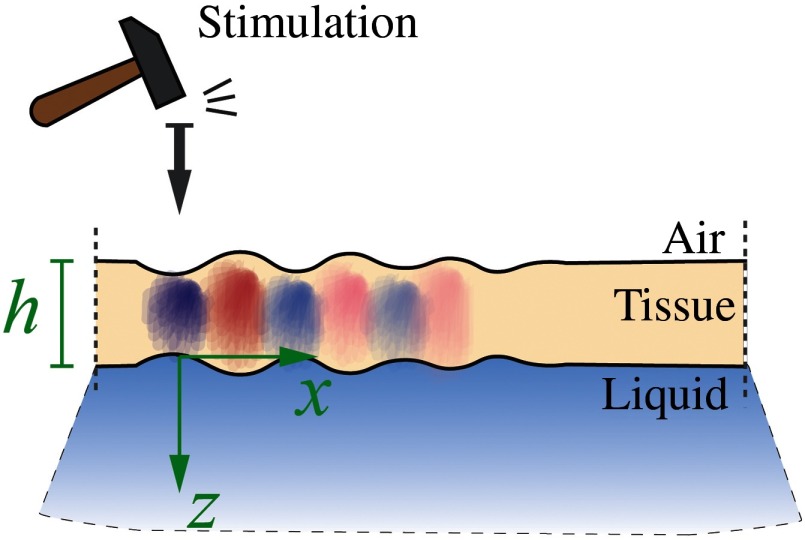
Guided mechanical waves propagate in a thin tissue layer bounded from one side by air and the other by liquid.

When a wave propagates in a medium with dimensions on the order of the mechanical wavelength, the medium becomes a waveguide. Tissue layer thickness, such as in the cornea, is typically on the order of a single mm, which for most dynamic OCE systems is on the same scale as the wavelength of an induced shear or surface wave. Thus, both the upper free boundary (air–cornea) and lower liquid-loaded boundary (cornea-aqueous humor) must be taken into account to accurately describe the propagation of guided waves in the cornea resulting from mechanical excitation at the air–cornea surface (Fig. 7).

The equations describing guided waves in a bounded layer (as a model of the cornea), even not taking into account its curved geometry (i.e., assuming a plate), are quite complicated; a simple equation such as those presented above for surface waves is not possible and only numerical solution reliably determines the modulus.

For an isotropic elastic material, the scalar potential ϕ and vector potential ψ cannot be omitted. A general form for a vertically polarized plain wave propagating in a plate in the x direction can be represented as

$$\phi_{1}=[A_{1}\;\exp(-q_{1}z)+A_{2}\;\exp(q_{1}z)]\exp[i(kx-\omega t)]\;\psi_{1}=[B_{1}\;\exp(-s_{1}z)+B_{2}\;\exp(s_{1}z)]\exp[i(kx-\omega t)],$$ (27)

where $q_{1}=\sqrt{k^{2}-k_{l}^{2}}$, $s_{1}=\sqrt{k^{2}-k_{s}^{2}}$, and $k_{l}=\frac{\omega }{C_{l}}$ $k_{s}=\frac{\omega }{C_{s}}$ are wave numbers for longitudinal and shear waves in soft tissue, respectively. Note that when the guided wave exists in plates of thickness smaller than the wavelength, the dominating wave modes are called Lamb waves.

In this case, boundary conditions can be written in the form

$${\sigma_{xz}^{\text{tissue}}|}_{z=-h}=0\;{\sigma_{zz}^{\text{tissue}}|}_{z=-h}=0\;{\sigma_{zz}^{\mathrm{liq}}|}_{z=0}={\sigma_{zz}^{\text{tissue}}|}_{z=0}\;{\sigma_{xz}^{\text{tissue}}|}_{z=0}=0\;{U_{z}^{\mathrm{liq}}|}_{z=0}={U_{z}^{\text{tissue}}|}_{z=0},$$ (28)

where h defines the plate thickness. Calculating the stress and displacement components from the scalar and vector potentials [Eq. (27)] and substituting them into boundary conditions [Eq. (28)], a 5×5 matrix characteristic equation is obtained

$$[\begin{array}{ccccc} 0 & -2ik^{2} & 2ik^{2} & -(k^{2}+s^{2}) & -(k^{2}+s^{2})\\ \rho_{l}\omega^{2} & -\rho_{T}\omega^{2}+2\mu k^{2} & -\rho_{T}\omega^{2}+2\mu k^{2} & -2i\mu ks & 2i\mu ks\\ 0 & \left(-\rho_{T}\omega^{2}+2\mu k^{2})\exp(kh\right) & \left(-\rho_{T}\omega^{2}+2\mu k^{2})\exp(-kh\right) & -2i\mu ks\cdot \exp(sh) & 2i\mu ks\cdot \exp(-sh)\\ 0 & -2ik^{2}\cdot \exp(kh) & 2ik^{2}\cdot \exp(-kh) & -(k^{2}+s^{2})\exp(sh) & -(k^{2}+s^{2})\exp(-sh)\\ 1 & -1 & 1 & i & i \end{array}]=0,$$ (29)

where $\rho_{l}$ and $\rho_{T}$ are densities of the liquid and tissue, correspondingly.

**Fig. 8:**
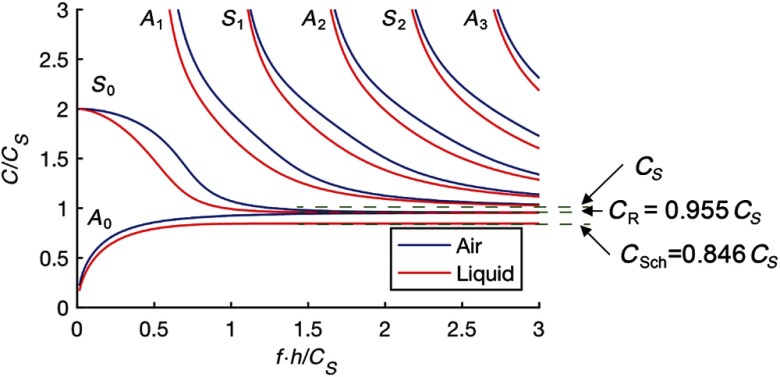
Phase velocity dispersion for propagating guided waves in a thin tissue layer loaded from one side by a liquid and the other side by air.

Equation (29) can be solved only numerically, resulting in the dispersion curves presented in Fig. 8, where the material is assumed to be nearly incompressible so that the longitudinal wave speed is much higher than the shear wave speed. These results can be compared to the blue curves showing the symmetric boundary condition case, i.e., tissue immersed in air, obtained using the same equation with $\rho_{l}=0$. As is evident from the plot, several modes can exist simultaneously. Most exhibit significant dispersion over a broad frequency band. Comparing the results for a water-loaded plate and free-boundary conditions, it is quite clear that the fluid has a significant influence on wave propagation. In Fig. 8, $A_{N}$ and $S_{N}$ indexes denote quasi- antisymmetric and symmetric Lamb modes. Note, however, that this notation is valid only for the symmetrical boundary case. If only a single side of the plate is water-loaded, neither symmetric nor antisymmetric modes exist. This fact is evident for the $A_{0}$ mode, which, for the case of tissue with free boundaries, has its high frequency limit equal to the surface wave speed, $C_{S}$. For single-sided water loading, the limit is the Scholte wave described in the section above. Note that an approximate solution represented in matrix form for a cylindrical geometry,^9^^,^^10^ and for the case of symmetrical boundary conditions of a water-immersed plate,^80^ has also been found and described in detail.

Here, we omit a detailed theoretical description of guided waves in the cornea because it is beyond the scope of this review, but note a few important conclusions from its finite thickness and liquid boundary condition. First, even when the material is purely elastic with no viscosity, guided modes are very dispersive. Simple measurement of the group velocity of propagating guided waves cannot be used to directly reconstruct the elastic modulus, E. Images based on the group velocity yield contrast related to elastic properties but may lead to serious quantitative error as the result clearly depends on the frequency range of group velocity calculation. To decode this complex wave behavior, dispersion analysis is required over a wide bandwidth. Consequently, a wideband mechanical wave source must be used to quantitatively estimate the elastic properties of the cornea from dynamic OCE measurements.

Second, wideband analysis of the experimental data can be used to estimate the high-frequency asymptotes of propagating guided waves, where the first asymmetric mode $A_{0}$ asymptotes to a Scholte wave with speed $C_{\mathrm{Sch}}=0.846C_{s}$, indicating a liquid load. Note that for the case of air, this limit corresponds to that for a Rayleigh wave. The first symmetric mode, $S_{0}$, has the high-frequency asymptote of a Rayleigh wave, $C_{R}=0.955C_{s}$, because it primarily propagates along the air–tissue interface. Other modes all asymptote to bulk shear waves propagating with speed $C_{S}$ in the bulk material with properties equivalent to that of the tissue. Thus, if the asymptotic behavior of propagating guided waves can be estimated accurately over a region of the bounded material, then the elastic modulus can be accurately computed in that region independent of the specific geometry, assuming that viscous effects are not significant. Tissue viscosity is considered in the next section.

#### 2.3.5 Tissue viscosity

As noted in Sec. 2.2, most soft tissues are viscoelastic. This means that at a specific mechanical frequency, the shear modulus μ (or equivalent Young’s modulus, E) is complex

$$\tilde{\mu }=\mu_{1}+i\mu_{2}.$$ (30)

As noted in Eq. (14), the real part ($\mu_{1}$) of the modulus is often called the storage modulus whereas $\mu_{2}$ is determined by viscosity. Therefore, equations for mechanical wave propagation in a bulk material should be corrected for tissue viscosity, i.e., μ should be exchanged with $\overset{˜}{\mu }$. Indeed, substitution of the complex value of $\overset{˜}{\mu }$ into the expression for the wave number, k, at a specific angular frequency, ω, (ω=2πf) gives

$$\overset{˜}{k}=\frac{\omega \sqrt{\rho }}{\sqrt{\overset{˜}{\mu }}}=\frac{\omega \sqrt{\rho }}{a+ib}=\frac{\omega }{a^{2}+b^{2}}(a-ib),$$ (31)

where

a=Real(μ˜)/ρb=Im(μ˜)/ρ. (32)

On the other hand, the complex phase of the propagating wave can be represented as

$$\overset{˜}{\varphi }=\omega t-\overset{˜}{k}z=\omega t-\frac{z}{C}+i\alpha z,$$ (33)

where

$$C=\frac{a^{2}+b^{2}}{a}\;\alpha =\frac{\omega b}{a^{2}+b^{2}},$$ (34)

are the phase velocity of the propagating wave and its attenuation coefficient, respectively. In general, they are frequency-dependent functions. Next, assume that functions C and α can be measured in an experiment. The parameters a and b can be determined as

$$a=\frac{\frac{\omega^{2}}{\alpha^{2}C}}{1+\frac{\omega^{2}}{\alpha^{2}C^{2}}}=\frac{C}{1+\eta^{2}C^{2}}\;b=C\frac{\alpha }{\omega }a=\frac{\eta C^{2}}{1+\eta^{2}C^{2}}.$$ (35)

Here, $\eta =\frac{\alpha }{\omega }$.

Finally, real, $\mu_{1}$, and imaginary, $\mu_{2}$, components of the shear modulus are calculated using frequency-dependent values of the phase velocity and attenuation coefficient

$$\mu_{1}=\rho (a^{2}-b^{2})=\rho C^{2}\frac{1-\eta^{2}C^{2}}{{\left(1+\eta^{2}C^{2}\right)}^{2}}\;\mu_{2}=2\rho ab=\frac{2\rho \eta C^{3}}{{\left(1+\eta^{2}C^{2}\right)}^{2}}.$$ (36)

Thus, both $\mu_{1}$ and $\mu_{2}$ can be determined from the measured frequency-dependent phase velocity and attenuation coefficient functions. When α=0, i.e., attenuation is absent, the propagating bulk wave is nondispersive (the phase velocity is equal to the group velocity and does not depend on frequency) and Eq. (36) reduces to the well-known bulk shear wave relationship (i.e., $\mu_{1}=\rho C_{S}^{2}$, Eq. 19).

When the attenuation coefficient is not zero, both components of the modulus are nonzero and generally frequency dependent because the stress–strain relation [Eq. (7)] has a finite time response. To describe this frequency dependence, specific models (e.g., Kelvin–Voigt or Maxwell) are commonly used to explicitly account for viscous damping. They are accurate only over a limited frequency range and, therefore, any derived parameters related to the elasticity and viscosity are only approximate descriptions of the viscoelastic characteristics of tissue. Nevertheless, frequency-dependent phase velocity and attenuation measurements can be used to extrapolate modulus estimates to zero frequency to obtain quantitative estimates of tissue elasticity.

**Fig. 9:**
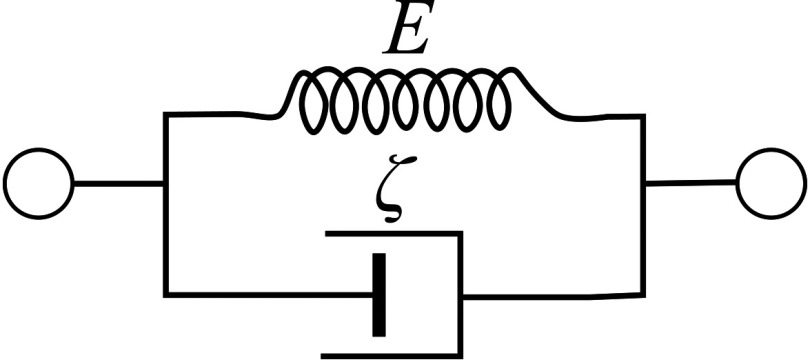
Schematic representation of Kelvin–Voigt model where E is the storage modulus and ζ is the viscosity.

To fully understand the effects of viscosity on OCE experiments in complex geometries such as the anterior segment of the eye, computational simulations are often employed. They require a specific viscoelastic model to capture the finite-time response of tissue subject to a dynamic load. The two most common are the Kelvin–Voigt and Maxwell models. The Kelvin–Voigt model^81^ assumes a viscous damper in parallel to a purely elastic spring (see Fig. 9).

Therefore, the stress–strain relationship can be represented as

$$\epsilon (t)=\frac{\sigma }{E_{1}}(1-e^{-t/\tau }),$$ (37)

where τ is the characteristic time of relaxation. When t→∞, Eq. (37) is equivalent to Eq. (11) used for static deformation.

Thus, in the Kelvin–Voigt model,^82^ the real part, $\mu_{1}$, of the elastic modulus is a frequency-independent constant ($\mu_{1}=\frac{1}{3}E_{1}$), whereas the imaginary part, $\mu_{2}$, is proportional to frequency

$$\overset{˜}{\mu }=\mu_{1}+i\omega \zeta ,$$ (38)

where ζ is the medium viscosity.

**Fig. 10:**
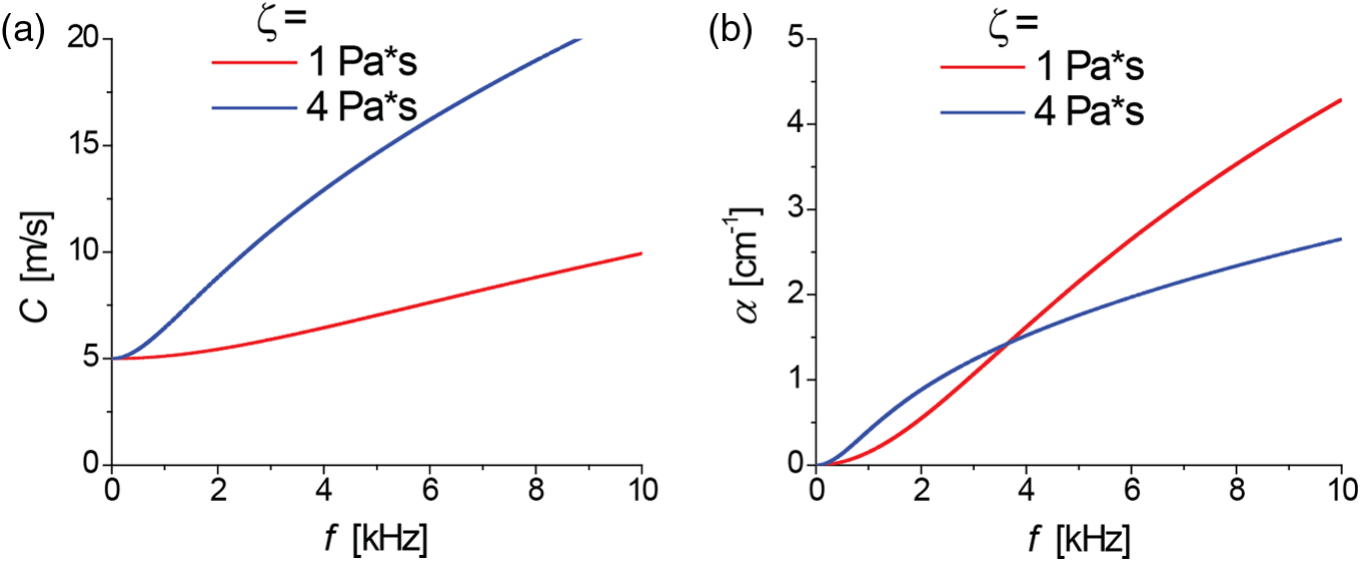
Frequency dependent (a) wave speed and (b) attenuation in the Kelvin–Voigt model. Parameters $\mu_{1}=25\;\mathrm{kPa}$ and ζ=1 and 4 Pa*s were used.

Substituting this relationship [Eq. (38)] into Eqs. (34)–(36), the dispersion curves for the shear wave speed and attenuation can be obtained

$$C(\omega )=\sqrt{\frac{2(\mu_{1}^{2}+\omega^{2}\zeta^{2})}{\rho (\mu_{1}+\sqrt{\mu_{1}^{2}+\omega^{2}\zeta^{2}})}}\;\alpha (\omega )=\omega \frac{\sqrt{\rho (-\mu_{1}+\sqrt{\mu_{1}^{2}+\omega^{2}\zeta^{2}})}}{\sqrt{2(\mu_{1}^{2}+\omega^{2}\zeta^{2})}},$$ (39)

which are illustrated for typical soft tissue values of viscosity, ζ, in Fig. 10 assuming $\mu_{1}=25\;\mathrm{kPa}$ (close to the shear modulus expected in human cornea at normal IOP). Clearly, even small viscosity (ζ=1  Pa*s gives ∼20% increase of propagation speed in the kilohertz range of frequencies) cannot be ignored.

In the limit of $\mu_{2}\ll \mu_{1}$, Eq. (39) reduces to

$$\underset{\mu_{2}\ll \mu_{1}}{\lim}\;C(\omega )\approx \frac{\sqrt{\mu_{1}}}{\sqrt{\rho }}(1+\frac{\omega^{2}\zeta^{2}}{4\mu_{1}^{2}})\;\underset{\mu_{2}\ll \mu_{1}}{\lim}\;\alpha (\omega )\approx \omega^{2}\frac{\sqrt{\rho }}{2\sqrt{\mu_{1}}}\frac{\zeta }{\mu_{1}},$$ (40)

which both have reasonable parabolic dependencies on frequency. However, this model can produce unphysical behavior at frequencies where the material with lower viscosity has higher attenuation coefficient. In general, the Kelvin–Voigt model can be reliably used only over a frequency range in which $\mu_{2}\ll \mu_{1}$ and the attenuation coefficient increases approximately quadratically with frequency.

**Fig. 11:**
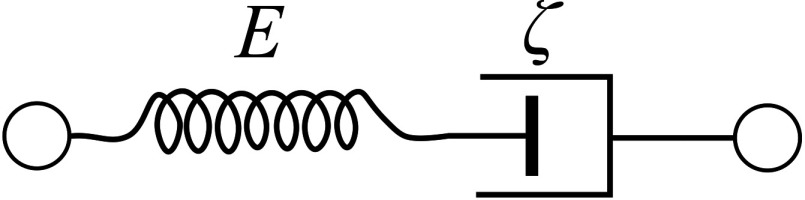
Schematic representation of Maxwell model.

The Maxwell model^83^ describes the material as a Newtonian fluid, when the elastic spring is in series with the viscous damper (see Fig. 11).

For this case, both real and imaginary parts of the shear modulus are frequency-dependent functions directly related to the time constant τ=ζ/E of the mechanical response

μ1=Real(μ˜)=ω2τ2ω2τ2+1μμ2=Im(μ˜)=ωτω2τ2+1μ. (41)

The phase velocity and attenuation using the Maxwell model are very different from those predicted by the Kelvin–Voigt model.^83^^,^^84^ Moreover, it was found that some tissues can be closely described by the Maxwell model whereas others by the Kelvin–Voigt over a finite frequency range, and other tissues by more sophisticated Zener or generalized Maxwell models.^81^^,^^83^^,^^84^^–^^88^ However, in general, neither Kelvin–Voigt nor Maxwell models correctly describe tissue viscoelasticity. In fact, it is very difficult to derive a general viscoelastic model to describe most issues and their pathologies over a wide frequency range.

To properly account for viscosity in computational simulations of OCE experiments, an appropriate viscoelastic model must be chosen to cover the experimental frequency range of wave propagation. Appropriately combining geometry-dependent and material-dependent dispersion in such simulations can be used to invert OCE measurements to obtain robust estimates of the real part of the modulus at zero frequency (i.e., tissue elasticity). Future computational studies must focus on such robust inversion of broadband mechanical wave measurements in different regions of the anterior segment of the eye.

### 2.4 Transient Excitation Sources

As described previously, a mechanical disturbance can be launched within a sample following an induced temporally short axial displacement at the tissue surface. Multiple excitation methods have been explored in OCE to launch a mechanical wave, including a physical actuator,^46^^,^^49^ ARF,^18^^,^^55^^,^^56^^,^^89^^–^^91^ air-puff,^50^^–^^53^ optical,^54^ and reflection-based ARF.^57^^,^^58^ The physical actuator can deliver a spatially compact, temporally short, indentation that can repeatedly launch a broadband mechanical disturbance. However, this contact method is highly undesirable in clinical ophthalmology due to patient discomfort. Herein, we describe less-invasive methods to launch mechanical waves in tissue.

#### 2.4.1 Acoustic radiation force

**Fig. 12:**
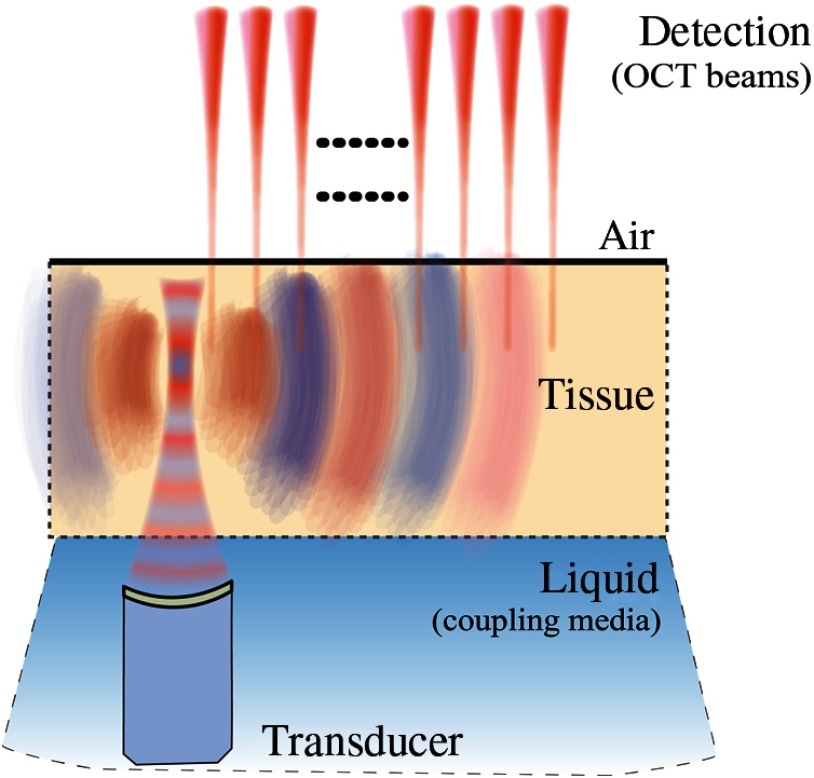
Schematic of shear wave excitation with ARF.

ARF delivered by an ultrasound transducer acoustically coupled to tissue has been used to induce displacements in various forms of elastography.^18^^,^^22^^,^^27^^,^^29^^,^^39^^,^^55^^,^^56^^,^^92^ US radiation force is commonly used in US-based elastography to remotely generate shear waves deep within tissue,^92^^–^^95^ and has been applied to OCE^89^^–^^91^ (see Fig. 12). The combination of US radiation force created by a conventional US probe with PhS-OCT to reconstruct quantitative cross-sectional maps of the shear modulus of tissue-mimicking phantoms has been demonstrated.^96^

ARF-based methods in elastography use loss and scattering mechanisms to convert acoustic energy into displacements.^97^^,^^98^ The ARF is determined by the spatial shape of the pump beam and duration of the ultrasound pulse.^93^^–^^95^^,^^98^ A very simplified, approximate expression for volumetric ARF based on acoustic absorption is

$$\mathrm{ARF}=\frac{2\alpha I}{C_{l}},$$ (42)

where I is the acoustic intensity of the pump beam, α is the ultrasound absorption coefficient, and $C_{l}$ is the speed of sound. ARF is usually noninvasive but still requires acoustic coupling.^93^^–^^95^^,^^98^

It is worth noting here that Nguyen et al. developed a pulse-compression technique that generates several ms bursts of ultrasound excitation in the megahertz range to modulate the force on a sample.^55^ The resulting displacements are then combined to simulate a broadband mechanical wave with high displacement amplitudes, but generated with low acoustic pressures. This method has potential clinical significance in ophthalmology for applications probing shear-waves at depth, for example, and can be used with any broadband source (e.g., air-coupled ultrasound). The ARF method also may be implemented with phased-array transducers to expand the imaging range of the system.^18^

#### 2.4.2 Air puff

**Fig. 13:**
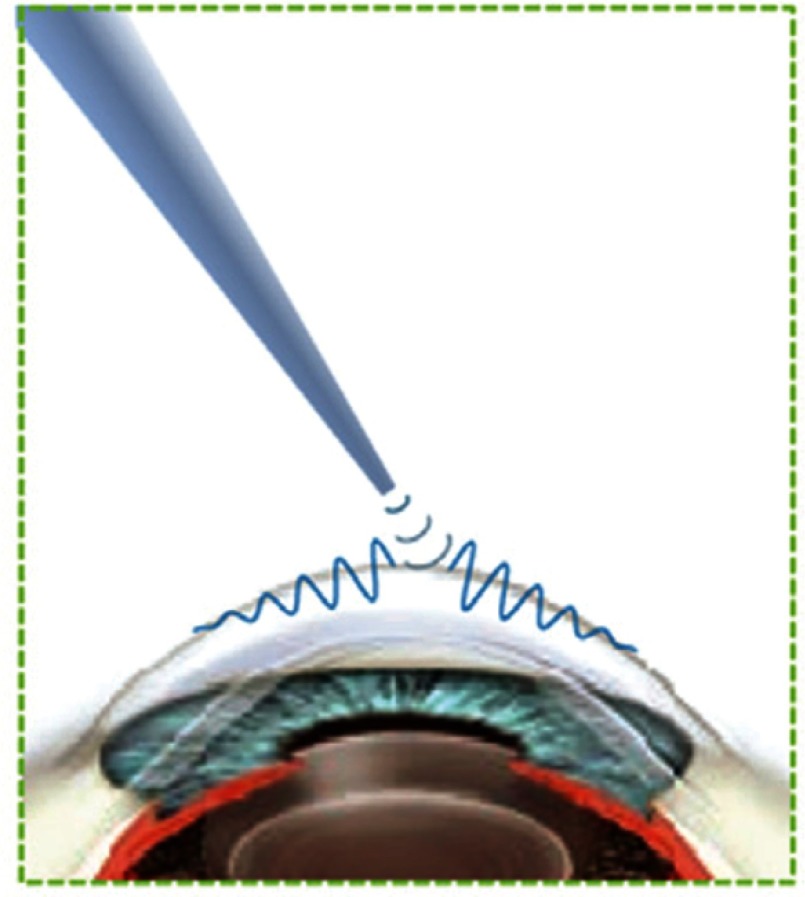
Diagram of shear waves excitation with a noncontact air-puff source. (Figure reproduced from Ref. 51 © Astro Ltd. Reproduced by permission of IOP Publishing. All rights reserved.)

For clinical applications of dynamic elastography, a totally noncontact system for generation/detection of mechanical waves is desirable and, in some cases (e.g., the eye), is required. Noncontact generation in soft media has been demonstrated in a limited number of studies. For example, an air puff (Fig. 13) was first demonstrated to produce mechanical waves in the cornea.^50^^,^^51^ This approach has certain limitations as the air-puff pulse can be very difficult to shape spatially due to spreading, resulting in low bandwidth mechanical waves. The air-puff method also suffers from slow relaxation times, limiting scanning configurations.

The air-puff method has shown promise in launching low-bandwidth mechanical waves that can be used to detect wave speed changes in corneal tissue at different ages,^52^ or following UV crosslinking^99^ based on measurements of group velocity.

Drawbacks of the air-puff are the low repetition rate, limited to about 100 Hz,^51^^,^^61^ which may limit the applicable OCT scanning methods (see Sec. 4). Additionally, difficulties with spatial shaping produce unreliable frequency content of the mechanical wave, resulting in measurement error of group velocity and dispersion. Air-puff excitation is noncontact, but the results obtained can provide quantitative maps of elastic properties only for highly controlled conditions at a coarse spatial resolution that may not be sufficient for applications in the anterior segment of the eye. Results obtained to date with air-puff excitation demonstrate the potential for quantitative elasticity analysis using noncontact systems, but to reliably translate noncontact methods into a routine clinical tool will most likely require more spatially localized and broadband mechanical sources, as discussed in the following sections.

#### 2.4.3 Pulsed laser excitation

**Fig. 14:**
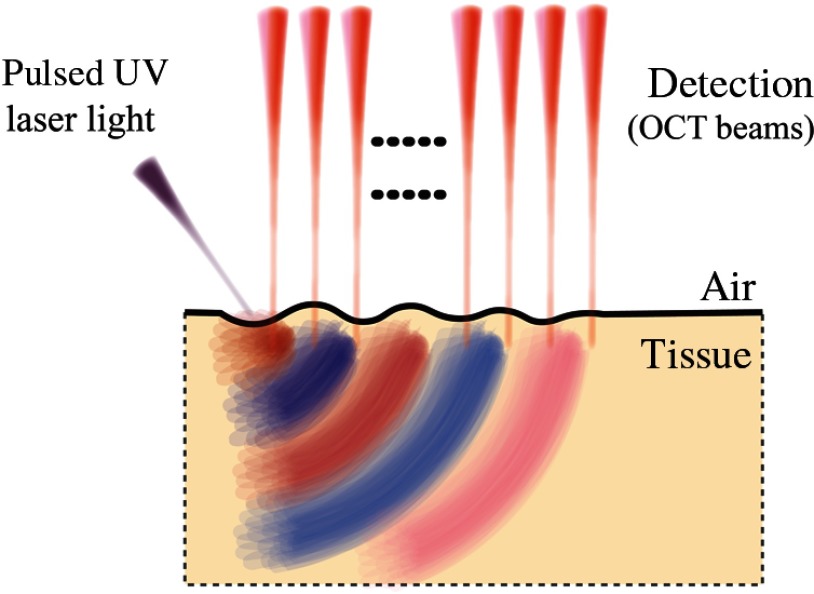
All-optical excitation and detection arrangement based on pulsed UV excitation.

An alternate noncontact approach has recently been demonstrated in which pulsed ultraviolet (UV) laser light is absorbed at the surface (within tens of μm depth) of the cornea to launch a mechanical wave^54^ (Fig. 14). The mechanical wave bandwidth can easily reach 10 kHz, providing full wave mode analysis and potentially superior lateral resolution in elasticity.^54^^,^^100^ The source can also be easily shaped with patterned laser excitation. Ultraviolet light is highly absorbed in soft tissue and is, therefore, a very efficient source of compressional acoustic waves. However, laser generation of shear displacement is detectable with current OCE devices only when the UV fluence approaches safety limits, which may be undesirable in clinical application.^100^

#### 2.4.4 Acoustic microtapping

**Fig. 15:**
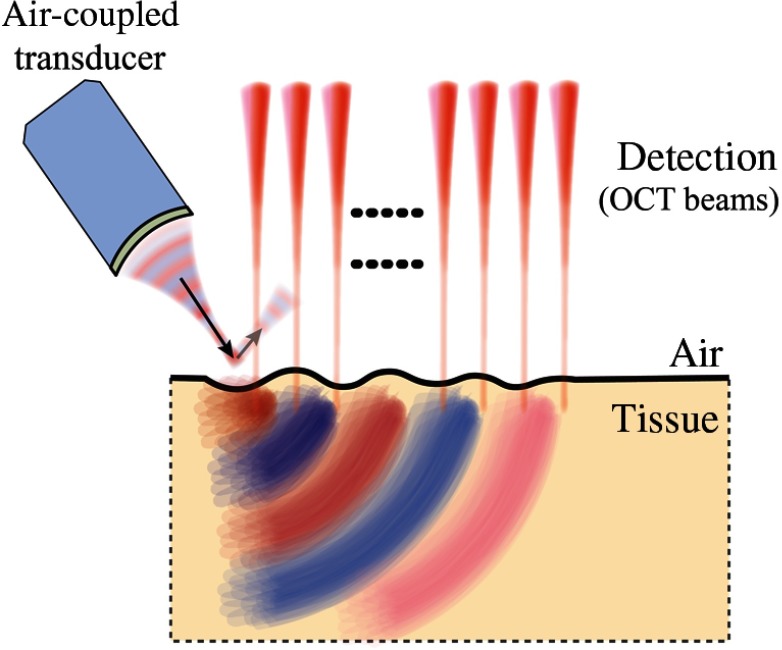
Air-coupled ARF (or AμT) for mechanical wave excitation in tissue.

Recently, a new technique called acoustic microtapping (AμT) was proposed,^58^ where a spatially and temporally sharp pressure is applied to the tissue surface via a focused air-coupled ultrasound transducer. This reflection-based approach converts acoustic energy to mechanical energy (reflection-based ARF) at an air/soft–medium interface (Fig. 15), launching a wave with nano to micrometer displacement amplitudes and sub-mm to micron-scale wavelength.^57^ Unlike relatively inefficient ARF techniques using acoustic loss and scattering mechanisms,^97^^,^^98^ a reflection-based approach can be a very efficient transducer of acoustic intensity into shear displacement.^58^

In reflection mode, the radiation pressure P (force per unit area) is

$$P=\frac{(1+R^{2})I}{C_{l}},$$ (43)

where R is the reflection coefficient at the air/medium interface, I is the acoustic intensity ($\text{Watts}/m^{2}$), and $C_{l}$ is the sound speed. For air-coupled ultrasound, the reflection coefficient at the air–tissue boundary is nearly one (R≅1) so that the radiation force can be approximated as $P=2I/C_{l}$. Since the sound speed in air is low (about 340  m/s) and nearly all acoustic intensity is converted into radiation pressure, significant force can be produced at modest acoustic pressures. The AμT source can be localized with the greatest portion of the energy at the focus,^58^ resulting in a temporally and spatially sharp noncontact method of excitation that shows great promise in reliable and robust generation of mechanical waves.

Reflection-based ARF of the pump beam (propagating in air) at the air/medium interface can produce surface transient displacements using acoustic frequencies in the ultrasound range (i.e., >20  kHz and up to several MHz) to generate propagating mechanical waves in soft tissue. AμT can also deliver a well-defined temporally sharp impulse, resulting in mechanical waves with a wide bandwidth. By shaping the pump ultrasound field launched with a focused air-coupled transducer, and also with acoustic masks, focusing mirrors^101^^–^^104^ and Fresnel plates,^104^^–^^106^ the radiation pattern (i.e., spatial distribution of I) can be manipulated at the air/medium interface. In addition, ultrasound arrays can electronically scan the pump field across the interface.

To establish shear wave imaging as a clinically useful tool, a reliable noncontact method must be demonstrated in a host of ophthalmic applications, a space that we believe is well suited to AμT.

#### 2.4.5 Bandwidth and wavelength of generated mechanical waves

Assume that an impulsive, spatially localized displacement is applied to a tissue surface. Independent of the source mechanism, the bandwidth of generated mechanical waves depends on the temporal and spatial characteristics of the excitation push. If the excitation is an infinitesimally short push, the mechanical wave bandwidth (BW) is determined by relaxation of the stress across the excitation beam, which occurs at the speed, $C_{s}$, of shear waves

$$\mathrm{BW}\approx \frac{C_{s}}{d},$$ (44)

where d is the size of the push cross section. Similarly, the characteristic wavelength of the generated mechanical wave is simply on the order of d.

Ultimately, the bandwidth/wavelength relationship of the mechanical wave determines the spatial resolution of reconstructed elastic modulus maps. Thus, for diagnostic mapping of elastic modulus, highly localized ARF is desirable. Additionally, field localization is limited by diffraction, so a high US frequency is desired. Increasing the pump US frequency, however, is limited by strong US attenuation in air (proportional to frequency squared).^107^^,^^108^ Note that AμT for mechanical wave excitation is very flexible in terms of the excitation beam pattern and can be adopted to a specific problem geometry.

An infinitesimally short pulse for mechanical wave excitation is actually not optimal. Indeed, to get the maximum pushing force, pump intensity should be confined during the time of stress relaxation across the excitation beam. Thus, the duration of the excitation push should be

$$T_{\text{push}}<\approx \frac{d}{C_{s}}.$$ (45)

**Fig. 16:**
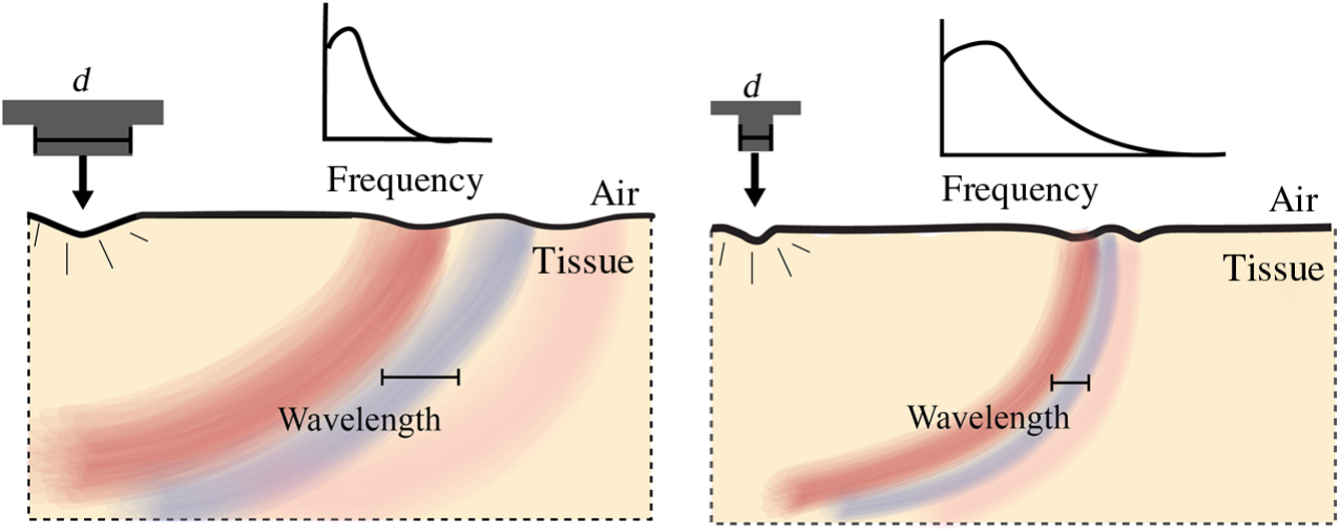
Wave characteristics from different push profiles on the same medium assuming the excitation time always satisfies Eq. (45).

A simple visualization of these relationships is presented in Fig. 16.

The push amplitude must be large enough to deliver an axial displacement that can generate a laterally traveling wave without damaging tissue. The resulting wave amplitude is determined primarily by the excitation force, but will be affected by local physiology as well. A stiffer tissue will have a smaller displacement magnitude for an equivalent excitation profile compared to a more easily deformable sample. The local tissue stiffness, in combination with OCT signal-to-noise ratio (SNR, Sec. 3), thus determines the necessary push strength, within safety limits, to launch a detectable wave within the sample.

## 3 Optical Coherence Tomography for Elastography

**Fig. 17:**
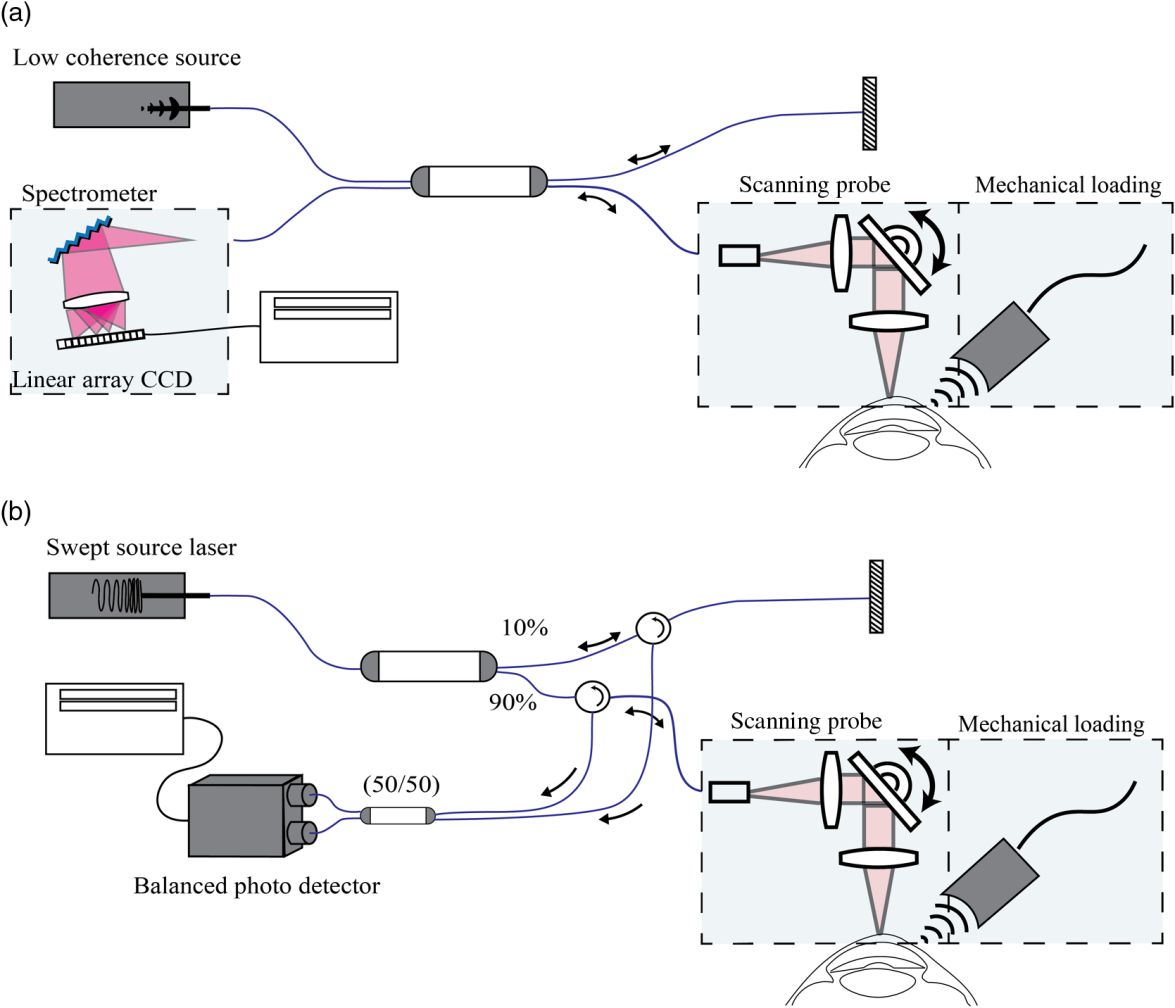
Schematic of OCE systems based on OCT implementation in the optical frequency domain: (a) SD-OCT and (b) SS-OCT.

OCT, based on the principle of optical interference, is well suited to measure tissue displacements related to local vibrations and strains. The detection methods and imaging protocols described within this section demonstrate how the OCT signal may be used to reconstruct high-resolution images of tissue elasticity based on the constitutive equations of the previous section. Because of the requirements of high sensitivity, fast image acquisition, and high phase stability, we summarize OCE methods based on Fourier domain, often termed spectral domain OCT (SD-OCT), or swept-source OCT (SS-OCT) configurations, as shown in Fig. 17.

In both configurations, the illumination source is split into two beams: one directed toward a stationary reference mirror and one toward the tissue sample. Light reflected by the corresponding interfaces is recombined by the coupler. In SD-OCT, a broadband light source and line-scan camera-based spectrometer is used to detect the optical fields as a function of wavenumber, k, to form a spectral interferogram. Note that while it is possible to detect the fields as a linear function of k,^109^^,^^110^ spectrometers commonly employed in SD-OCT require a digital rescaling of wavelength (λ) into wavenumber (k), resulting in the loss of signal sensitivity at increasing depths.^111^^,^^112^ Figure 17(b) demonstrates an alternative configuration utilizing a swept frequency laser source. In SS-OCT, backscattered light passes through optical circulators and is recombined by a 2×2 coupler for detection by a balanced photodetector with even sampling in k-space, often resulting in improved signal sensitvity.^113^^–^^115^ In either configuration, the spectral interference fringes at the detector may be expressed as^46^

$$i(k)=\frac{1}{2}\phi S(k)\delta k\{R_{R}+\underset{n}{\sum }R_{n}+2\sqrt{R_{R}}\underset{n}{\sum }\sqrt{R_{n}}\;\cos[2k(z_{n}+\delta z_{n})]\},$$ (46)

where S(k) is the source power spectral density, k is the wave number, δk is the spectral resolution of the spectrometer in SD-OCT or the linewidth of the light source in SS-OCT, ϕ is the detector sensitivity, $R_{R}$ is the reference arm reflectivity, and $R_{n}$ is the reflectivity of the sample at depth $z_{n}$, where $z_{n}$ is physically described as the integer multiple of the discrete sampling interval in the z-direction (determined by mπ/δk, where m is the positive or negative integer of the A-scan array). The $\delta z_{n}$ term arises from subresolution deviations in position of the local scatterer from the signal at the discrete location, $z_{n}$. In SD-OCT, i(k) is the integration over the A-scan exposure time while in SS-OCT i(k) is instantly sampled by a high-speed photodetector. In either case, a discrete Fourier transformation of i(k) is performed to give the depth-encoded complex signal defined by

$$I(z_{n})=\Gamma (z_{n})\exp(jk_{0}\delta z_{n}),$$ (47)

where $\Gamma (z_{n})$ describes the unity-amplitude z-domain autocorrelation function, and $k_{0}$ is the center wavenumber of the source. We note that this representation is simplified for the purpose of this review, and refer the reader elsewhere for a more detailed description.^115^ The integer step ($\Delta z_{n}$) in the z-domain autocorrelation function (often referred to as the coherence function) defines the axial resolution in OCT.

Due to the Fourier relationship between the source power spectral density and the electric field, the axial resolution in free space may be defined by the coherence length of the recombined light, which is related to the spectral bandwidth of the light source as^115^

$$\frac{l_{c}}{2}=\frac{2\sqrt{\ln(2)}}{\Delta k}=\frac{2\;\ln(2)}{\pi }\frac{\lambda_{0}^{2}}{\Delta \lambda }.$$ (48)

For wavelengths typically used in biological applications, the axial resolution is roughly in the micrometer scale. Note that axial resolution is decoupled from lateral resolution. Lateral resolution in OCT is determined by the center wavelength and the numerical aperture of the objective lens.

The fundamental properties of the OCT signal may be used in OCE, but it is worth noting that differences in scanning protocols and imaging contrast mean that OCE and OCT capabilities are not identical. Motion detection in OCE, for example, requires carefully timed image acquisitions that are postprocessed to infer changes in tissue scatterer location and naturally reduce the resolution compared to structural OCT. The following section describes how changes in the depth-encoded complex signal, [$I(z_{n})$], may be used to reconstruct high-resolution images of ocular tissue elasticity based on the constitutive equations described in the previous section, providing examples of current techniques and future directions.

### 3.1 Speckle Tracking

Akin to early-stage ultrasound elastography, speckle tracking was the primary technique to measure displacement in early OCE studies, mostly in static OCE.^1^ The coherent nature of interferometric techniques used in OCT creates constructive and destructive interference patterns giving the final image its grainy appearance, referred to as speckle. When optical scatterers within a defined region translate, assuming their relative positions remain constant, the imaged speckle translates according to local scatterer motion and may be used to map displacement within a sample.^116^^,^^117^ Speckle tracking estimates tissue motion by comparing unloaded and loaded image reconstructions; a reference OCT image is taken and a subsequent image following an induced stress is used to infer strain. In particular, displacement is determined from the maximum crosscorrelation of a multipixel kernel in subsequent cross-sectional images within the same tissue region. The basic concept is presented in Eq. (49) for a 2-D case considering a single scattering element within a sample at location (x,z).^1^

$$CC(U_{x},U_{z})=\frac{\int_{-Z/2}^{Z/2}\int_{-X/2}^{X/2}I_{1}(x,z)I_{2}(x-U_{x},z-U_{z})dx\;dz}{\sqrt{\int_{-Z/2}^{Z/2}\int_{-X/2}^{X/2}I_{1}^{2}(x,z)dx\;dz\int_{-Z/2}^{Z/2}\int_{-X/2}^{X/2}I_{2}^{2}(x-U_{x},z-U_{z})dx\;dz}},$$ (49)

where I is the imaged intensity at a single pixel element (i.e., real $\left[I(z_{n})\right]$), X and Z define the local window of interest, and $CC(U_{x},U_{z})$ is the correlation coefficient for the displacement components $U_{x}$, $U_{z}$. When a compressive load or mechanical wave deforms the object, a single scattering element relocates to a position defined as ($x+U_{x}$, $z+U_{z}$). Translational motion, defined by the corresponding displacement vector $\overset{\to }{U}=(U_{x},U_{z})$, may be detected between OCT B-scans at each pixel by solving for the displacement vector that maximizes the normalized crosscorrelation term within the defined window size, X and Z, assuming speckle remains correlated. The maximum correlation gives the most likely location of the corresponding scatterer within a predetermined window and may be readily expanded into 3-D.^118^^,^^119^ To map a displacement vector to each image, the correlation window is moved across the B-scan image and the process repeated at a number of locations. In vibrational or dynamic OCE, multiple sequential image acquisitions are acquired and the displacement vector may be tracked temporally to measure vibration speed or detect wave propagation.

An advantage of speckle tracking is that both lateral and axial displacements can be detected. This may be advantageous in fully describing the 3-D stress/strain behavior within tissue, potentially allowing alternative loading and scanning schemes. The ability to detect lateral and axial motion may also be beneficial when detecting shear waves that travel into tissue at an angle, vibrating in multidimensions.

For static deformations, the displacement map may be used to derive the differential strain within a defined region. As noted in Sec. 2, components of the symmetric strain tensor can be derived using spatial derivatives of the displacement vector. For example, the two longitudinal strains within a specific plane acquired from 2-D speckle tracking can be written as

$$\epsilon_{zz}(x,z)≅\frac{\Delta U_{z}(x,z)}{\mathrm{dz}},\;\epsilon_{xx}(x,z)≅\frac{\Delta U_{x}(x,z)}{\mathrm{dx}},$$ (50)

where dz and dx represent the region over which the displacement vector is considered. Strain measurement accuracy may be improved by averaging over a larger dx or dz, at the expense of spatial resolution, but remains susceptible to uncertainty due to error in both displacement and pixelated intensity terms.^117^

Sensitivity to motion is governed by the relationship among image speckle size, image pixel size (as determined by the optical arrangement), and decorrelation statistics, as a corresponding scatterer must move a sufficient distance for speckle motion to be imaged at a neighboring pixel.^120^^–^^122^ In general, correlation-based speckle tracking can track motion exceeding a quarter of the OCT laser wavelength without ambiguity. Thus, the displacement map resolution is usually a few times that of OCT, determined by the chosen pixel kernel and on the order of micrometers. In strain maps, this resolution is even worse. The upper limit of detectable motion is determined by speckle correlation. When subject to high stress, and subsequently large deformations, the relative positions of light scatterers will reorganize and cause severe speckle decorrelation between frames, rendering speckle-tracking methods unusable. Fast scanning rates may be implemented to ensure speckle remains correlated between B-scans under high strain-rates. More advanced processing algorithms have been proposed to track speckle displacements via the OCT intensity signal, many of which trade off sensitivity with dynamic range.^116^ Parametric methods have been demonstrated in OCE with displacement sensitivity as low as 0.1  μm, but with a dynamic range of ±0.8  μm. For example, 3-D speckle tracking was shown to achieve a displacement sensitivity of, at best, 0.3  μm in phantoms, but was very computationally intense.^123^ Dynamic OCE thus requires large (potentially unsafe) excitation pressures to generate displacements large enough to be detected using speckle tracking methods. Alternatively, the detectable strain range in static OCE is limited by the low dynamic range.

Traditional speckle-tracking methods require relatively large tissue displacements which may present challenges when applied to sensitive human tissue such as the cornea. Additionally, subresolution motion often causes speckle to “blink” or “boil” as a function of the phase-sensitive rearrangement of scatterers within a pixel that modulates the intensity.^117^ Speckle variations are especially pronounced for deformations of scatterers partially moving in/out of the OCT scan plane. Also, both in-plane and out-of-plane strains can redistribute scatterers, leading to fast rates of speckle decorrelation.^48^^,^^118^

Speckle decorrelation is a major source of error in displacement measurements, producing low SNR estimates of tissue strains, especially for smaller strain values.^117^ Subresolution displacement sensitivity may be achieved by correcting for intensity error induced by subresolution changes in the phase term.^124^ Additionally, many of the likelihood estimator equations used to track tissue motion based on speckle patterns are computationally intensive and very time consuming, making real-time rendering of tissue strain difficult.

### 3.2 Phase-Sensitive OCT

Similar to speckle tracking, PhS-OCT may be used to detect motion by analyzing the differential signal between successive scans following, or during, an applied load. The phase term of each complex signal, while random in nature, is exploited between successive scans to detect subresolution motion within a sample.^36^^,^^46^^,^^115^ For each scan location, the phase term may be defined as

$$\varphi_{\mathrm{opt}}(x,z)=im[I(z_{n})]=im[E(z_{n})\exp(jk_{0}2\delta z_{n})].$$ (51)

As subresolution deviations from the z-location ($\delta z_{n}$) define the relative position of $\varphi_{\mathrm{opt}}(x,z)$, the phase will remain constant if scatterers located at (x,z) remain stationary. If the particle vector displacement under load deviates axially, the phase term will shift according to

$$\Delta \varphi_{\mathrm{opt}}(x,z,t)=2n\overset{¯}{k}\Delta U_{z}(x,z,t),$$ (52)

where n is the refractive index of the scattering component, and $\overset{¯}{k}$ is the average wave number. Equation (52) may be rearranged to readily solve for $\Delta U_{z}(x,z,t)$ by

$$\Delta U_{z}(x,z,t)=\frac{\Delta \varphi_{\mathrm{opt}}(x,z,t)\overset{¯}{\lambda }}{4\pi n},$$ (53)

where $\overset{¯}{\lambda }$ is the average wavelength, and $\Delta U_{z}$ is the displacement between scans. In static OCE, the displacement vector may be directly related to strain. Thus, spatially and temporally resolved strain and displacement maps may be generated using subenvelope displacements, greatly improving motion sensitivity compared to speckle tracking.

The minimum detectable change in phase, and thus the minimum detectable displacement amplitude, is determined by system noise and inversely related to SNR (i.e., 1/SNR).^125^ This expression has been shown to hold in practice over the range of typical OCT SNRs, from 20 to 50 dB, as a quick estimate of displacement sensitivity. The actual motion sensitivity of phase-sensitive methods is more complex and has recently been characterized by Xu et al.^126^ It also has been reported that phase decorrelation at small displacements affects minimum displacement measurement.^127^ Nevertheless, the minimum detectable displacement amplitude is typically in the nanometer to subnanometer range for micron wavelength light sources.

It follows that the upper range of measurable displacement is determined by the change in detected phase between each scan. If the scatterer travels far enough to induce an absolute value greater than π phase shift, phase wrapping occurs. Discontinuities in the phase shift can be corrected using phase-unwrapping algorithms, where corrections of up to five wrapping discontinuities have been reported.^32^ In practice, induced strain amplitudes falling within the nm range do not produce significant phase wrapping and can be detected using PhS-OCT, providing superior sensitivity to speckle-tracking methods.

In dynamic OCE, the displacement term is defined as the change in scatterer position between scans and is more appropriately reported by the depth-resolved vibration speed^35^

$$U_{zt}=\frac{\Delta \varphi_{\mathrm{opt}}(x,z,t)\overset{¯}{\lambda }}{4\pi nf_{s}^{-1}},$$ (54)

where $f_{s}$ is the scan rate. The vibration speed may be spatially and temporally mapped for dynamic OCE applications, enabling fast detection of a mechanical disturbance induced at relatively low stress. Further, $U_{zt}$ can be related to the spatially resolved strain rate by

$$\epsilon_{zz}^{\prime }(x,z,t)=\frac{\Delta U_{zt}(x,z,t)}{z_{0}}=\frac{\Delta [\Delta \varphi_{\mathrm{opt}}(x,z,t)\overset{¯}{\lambda }]}{4\pi nf_{s}^{-1}z_{0}},$$ (55)

where $\Delta U_{zt}(x,z,t)$ is the change in vibration speed over range $z_{0}$ centered at position (x,z). Equation (55) can be easily related to the absolute strain by integrating over the time duration between scans as

$$\epsilon_{zz}(z)=\overset{T}{\underset{0}{\int }}\epsilon_{zz}^{\prime }(x,z,t)dt=\overset{T}{\underset{0}{\int }}\frac{\Delta [\Delta \varphi_{\mathrm{opt}}(x,z,t)\overset{¯}{\lambda }]}{4\pi nf_{s}^{-1}z_{0}}dt.$$ (56)

Clearly, PhS-OCT can characterize the displacement, vibration speed, strain, and strain rate at a theoretical resolution close to that of OCT.

**Fig. 18:**
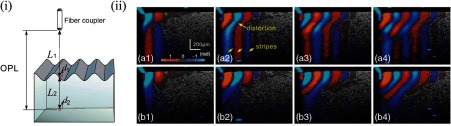
The OPL of incident light must account for changes in refractive index that delay the measured signal. The surface ripple, (i) for example, would result in error in displacements measured at a specific depth. (ii) Dynamic displacements within a phantom are shown without (a1–a4) and with (b1–b4) motion. (Figure reproduced from Ref. 46.)

As changes in phase accumulate in depth, any change in the optical path length (OPL) of the detected signal would complicate depth-resolved displacement detection. That is, motion within tissue layers (with different refractive indexes) closer to the sample surface would add to the apparent motion of tissue below. Phase measurements must thus account for sample surface motion and refractive index mismatch within each layer.^46^^,^^128^ When a shear wave is excited from the surface of a tissue, that surface experiences a ripple that manifests as a change in phase across all depths within the sample. Therefore, a motion artifact compensation algorithm is often incorporated to remove surface ripple effects, as demonstrated by Eq. (57) and Fig. 18, where $\varphi_{d}$ is the detected phase, $\Delta \varphi_{\mathrm{surf}}$ is phase at the sample surface, and $\Delta \varphi_{c}$ is the corrected phase at a z-location^46^

$$\Delta \varphi_{c}(z,t)=\Delta \varphi_{d}(z,t)+(n_{2}-n_{1})\Delta \varphi_{\mathrm{surf}}(t).$$ (57)

For SS-OCT, one additional source of noise comes from optical frequency jitter common in swept-wavelength light sources. An unstable frequency component may manifest as motion within the sample and thus must be addressed. Frequency jitter noise can be overcome with optical frequency referencing techniques, such as using a fiber Bragg grating^129^ or a Mach–Zehnder reference interferometer to perform spectral recalibration for more precise triggering.^42^^,^^49^^,^^115^ Environmental noise has also been reduced by utilizing a common-path interferometer.^32^ For high-speed Fourier domain mode-locked (FDML) laser sources, special scanning protocols have been proposed to compensate for interbuffer instability.^100^ A practical approach to improve SNR in OCE with low amplitude displacements is to utilize a coded excitation method, as demonstrated by Nguyen et al.^55^ For subsurface axial strain measurements, the SNR can also be improved by linearly fitting the displacements over a small region to improve the accuracy and robustness of compression-based strain estimation.^34^

Additional phase noise is introduced by spatial misalignment of A-scans. This may be corrected using high lateral spatial sampling.^125^ Similar phase instabilities may be introduced by mechanical instabilities within the system, such as that introduced by vibrations in the scanning mirror.

### 3.3 Hybrid Methods

There are a growing number of alternative motion detection schemes being explored in OCT, including intensity-based Doppler variance,^130^^,^^131^ displacement measurement based on the amplitude of the complex correlation coefficient,^118^ and hybrid estimation of phase and intensity displacement tracking.^124^ These recent approaches often utilize both intensity-based correlation techniques and phase-sensitive methods to track axial and lateral displacements. A limitation, however, is that these processing algorithms often require larger displacements or averaging over a number of pixels.^131^ Regardless, new motion detection mechanisms in OCT could provide additional information on the strain response in all 3-D, providing potentially useful information to calculate elastic moduli.^132^

## 4 OCE Methodology

In this section, we describe scanning protocols and reconstruction methods used to generate 2-D and 3-D maps of mechanical properties based on OCT motion detection. We touch on practical issues related to OCE resolution and the importance of system design, providing examples of current techniques and progress.

### 4.1 Static/Quasistatic Imaging

**Fig. 19:**
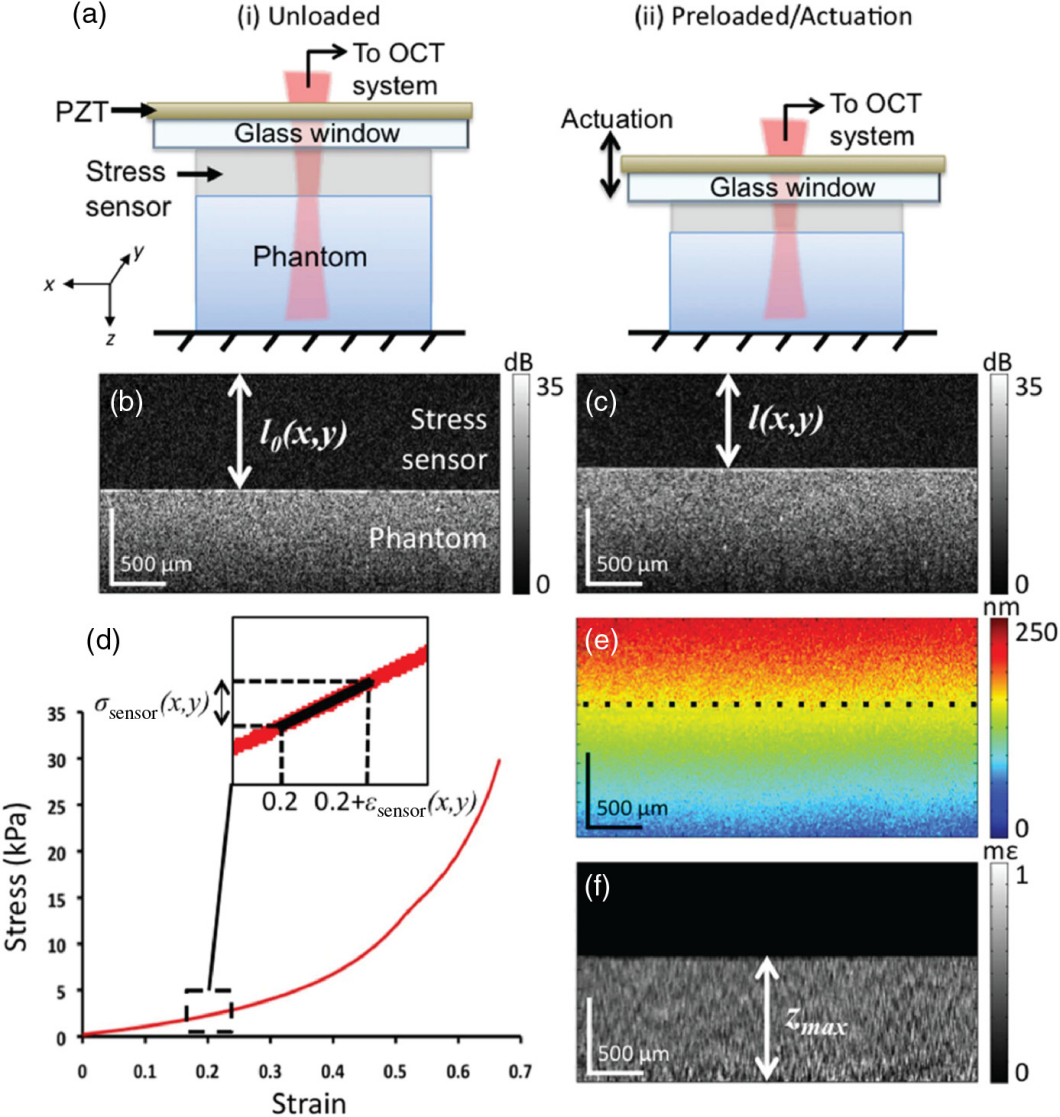
Schematics demonstrating static compression OCE, where displacement and strain maps are generated from (a) (i) unloaded and (ii) preloaded sample. The resulting OCT image in the (b) unloaded and (c) preloaded case with the corresponding schematic. The known stress–strain curve of the stress sensor used to quantify E is shown in (d). The displacement map is shown in (e) and the strain map in (f). (Figure reproduced from Ref. 34.)

An example of the working principal of static deformation OCE is shown in Fig. 19. A flat-plat actuator applies an axial load to the sample and PhS-OCT is used to generate displacement maps [Fig. 19(e)] that may then help infer mechanical properties. In this example, a stress sensor is used to quantify the stress tensor at the phantom surface so the physical modulus may be estimated. A similar configuration was recently demonstrated on the human cornea to map local displacements following an induced stress applied with a modified goniometer.^48^

The axial stress in this case is not time-dependent and thus the scanning protocol has very few requirements: simply “before and after” images are needed to measure strain. The lateral resolution of strain detection is determined by the B- and C- scan density across the imaging window and is roughly determined by the objective lens. The axial resolution is determined by the area over which the strain is measured; thus, the final elastogram resolution is roughly multiple times that of the typical OCT axial resolution. Rigorous analysis of noise and decorrelation in speckle tracking yields a maximum reliable strain resolution estimate of 20 to 30 pixels.^117^ Phase-sensitive methods have similar limitations because strain computation uses multiple axial displacement measurements.^127^

### 4.2 Dynamic Imaging

Detecting vibrational and mechanical wave motion requires fast scanning and accurate timing. For vibrational analysis, the scan speed is determined by the Nyquist sampling theorem, the size of the imaging region, and the frequency of vibrations used to probe the sample. In mechanical wave imaging, scan requirements are more complex. For example, a wave traveling at 5  m/s will take 1 ms to cross an imaging range of 5 mm (note: corneal wave speeds tend to be between 1 and 15  m/s).^58^^,^^133^ To have truly microscale OCE resolution of, say, 100  μm, the wave must be captured at no fewer than 50 locations. To capture a 5  m/s wave at 50 locations over a 5-mm region, a frame rate of at least 50 kHz is necessary. As wave speeds in corneal tissue higher than 5  m/s are common, a clinically practical OCE system may even require frame rates in excess of 50 kHz. Historically, the limitation of scan speed has hindered OCE development. However, recent developments in system design have allowed OCE imaging to approach practical requirements.

#### 4.2.1 M-B imaging

**Fig. 20:**
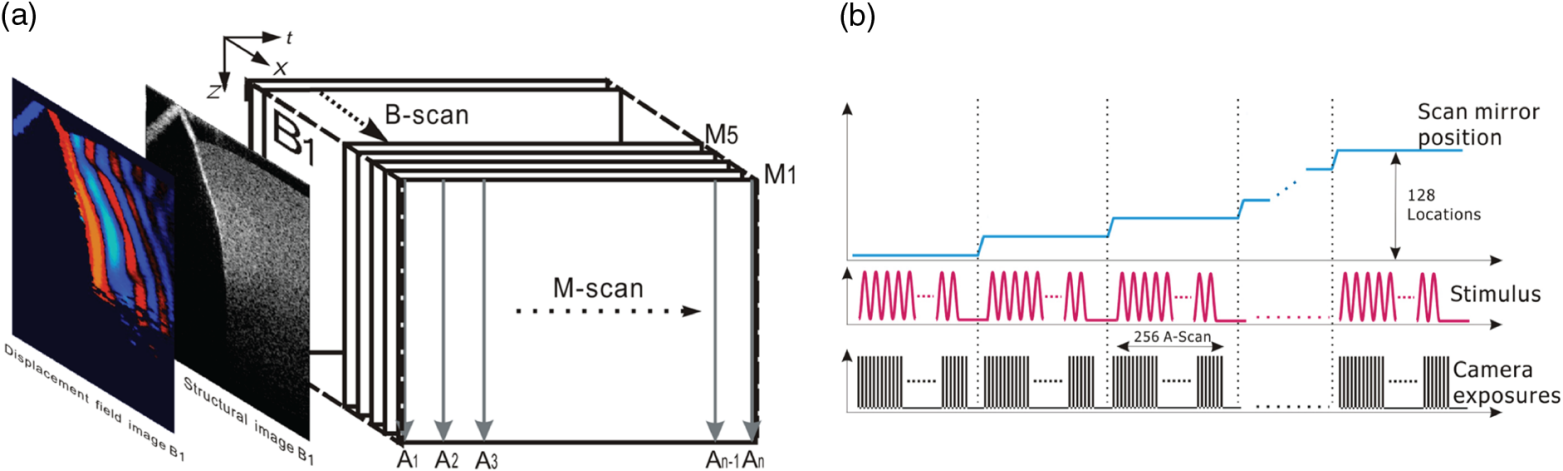
(a) M-B scan protocol. (Figure reproduced from Ref. 47) (b) Timing schematic of scanning protocol (Figure reproduced from Ref. 46.)

Similar to ultrasound elastography based on ARF scanning,^134^ M-B scan protocols may be used in OCE to achieve effective frame rates in the tens of kHz range. Conventional M-mode imaging repeats axial scans at a fixed location to achieve dynamic imaging with the highest temporal resolution allowed by the imaging device (A-scan rate). By repeating targeted dynamic events (loading scheme), the M-B scan protocol can provide effective high frame rates to track dynamic processes in the space-time domain. The M-B scan method, demonstrated by both Song et al.^46^^,^^47^ (Fig. 20) and Wang and Larin,^40^ utilizes multiple excitations to collect repetitive A-scans (M-mode scans) at multiple spatial locations then matched in time to generate B-frames for effective data collection fast enough to capture mechanical wave propagation at multiple locations within a sample. The technique has been demonstrated with an equivalent frame rate of up to 92 kHz.^135^

**Fig. 21:**
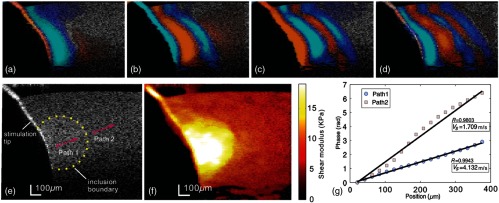
Modulus estimation based on shear wave propagation using M-B scan protocol. (a)–(d) demonstrate propagating mechanical waves at different time frames. The structural OCT image (e) shows the boundary of a stiff phantom inclusion and the paths used to calculate wave speed. Map of the coarse estimation of modulus is shown in (f) and the wave speed measurements shown in (g). (Figure reproduced from Ref. 47.)

An example of how tissue elasticity information can be obtained using local group velocity estimates of the propagating mechanical wave is shown in Fig. 21. Here, the group velocity was inverted to produce a coarse estimate of the shear modulus. Even with this simple approach, phantom regions with different elastic modulus can be easily visualized. A problem, however, is that multiple M-mode scans per B-scan subjects the sample to multiple transient excitations per image. Such excessive “tapping” on surfaces such as the cornea may be less than ideal for clinical applications and may actually result in error when imaging tissues with long relaxation times. Repeated tissue excitation remains a limitation that calls for even faster scan protocols.

Still, early-stage M-mode imaging has been utilized to measure the frequency response of corneas,^71^ and has shown promise in elucidating corneal anisotropy^133^ and in assessing the effects of corneal UV crosslinking.^75^^,^^136^

#### 4.2.2 B-M scan high-speed imaging

**Fig. 22:**
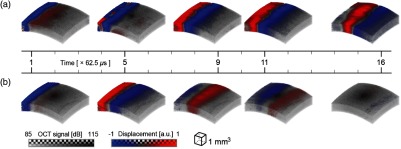
FDML system demonstrating the ability to spatially and temporally capture a propagating mechanical wave in porcine cornea. (Figure adapted from Ref. 58—see movies related to these measurements in the same publication.)

Recent developments in swept-laser source configurations have greatly improved the scanning speeds possible in OCT. High frame rate scanning is now possible with B-M configurations imaging wave propagation following a single transient excitation, where consecutive B-scans are acquired and used to detect motion within the 2-D cross section. A-scan rates as fast as 1.5^137^ and 1.628 MHz^100^ have been reported using an FDML laser source and a single element detection scheme. Increased scan rates make four-dimensional (4-D) (three in space plus time) volumetric data collection feasible for clinical applications. 4-D tracking of waves in porcine cornea at different IOP has been demonstrated by effectively scanning the excitation and detection to generate a C-scan in the third dimension (Fig. 22), with total data collection in under 100 ms.

Other scanning methods have been proposed to improve temporal and spatial resolution for clinically focused systems.^138^^–^^141^ Full-field-OCT methods have been demonstrated in phantom samples, but are limited to enface imaging, rendering 3-D imaging more difficult.^139^ Polarization-sensitive systems have been used to assess both strain and birefringence.^142^ Improved spatial resolution has been reported using parallel acquisition channels to simultaneously capture M-mode data at multiple spatial locations,^143^ or through multiple (less than 10) excitations with temporal scans at different locations, combining M-B mode with B-M mode scanning protocols. The tradeoff is that the total image acquisition time is lengthened.

**Fig. 23:**
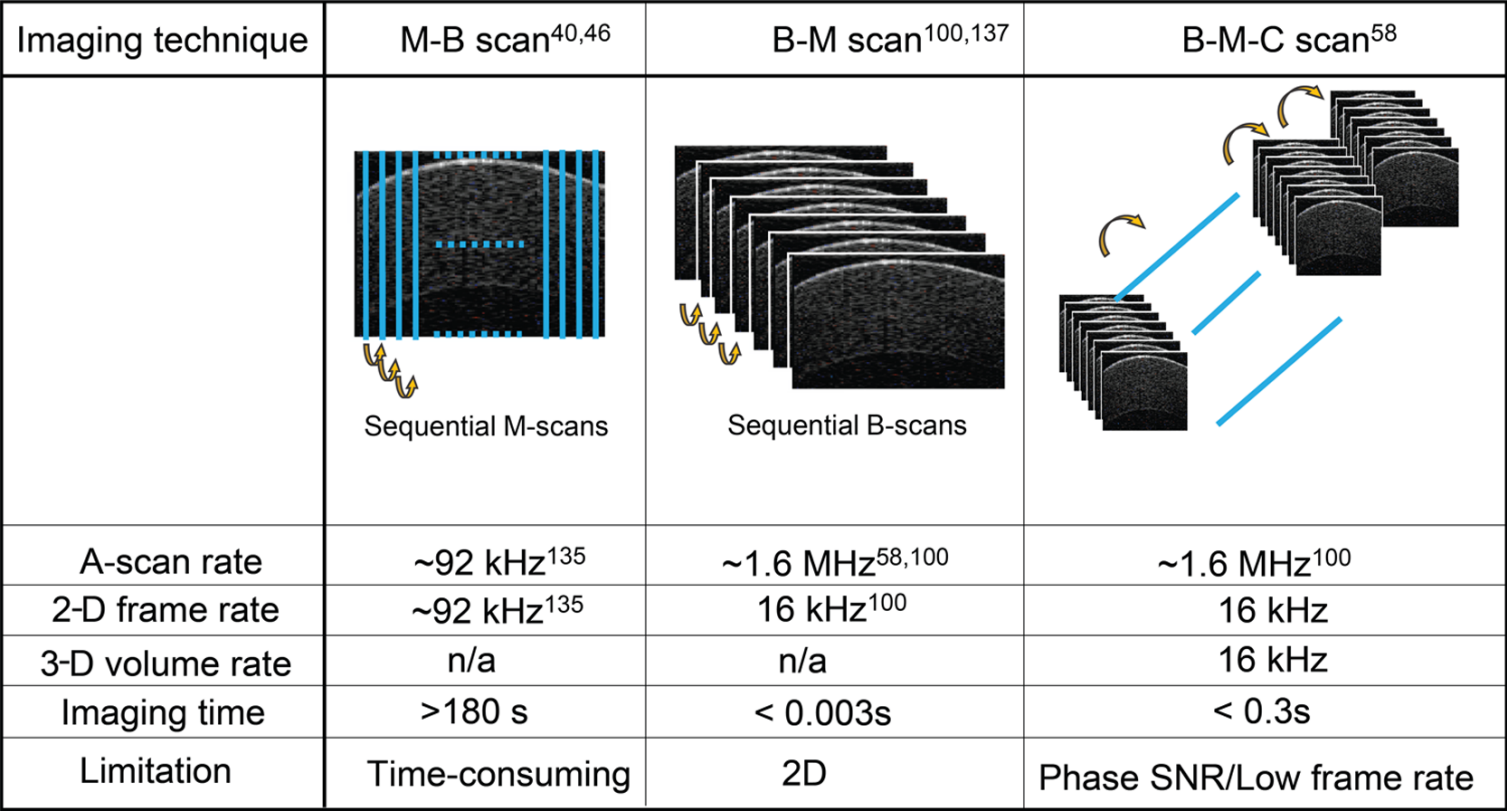
Summary of common scan protocols reported in OCE.

While high-speed OCE scanning (summarized in Fig. 23) shows great promise in mapping wave properties with a resolution on the order of a couple of hundred microns, scan speeds are not yet sufficient to capture most wave speeds in tissue to generate microscale resolution images. To return to the previous example where a 5  m/s wave travels 5 mm in 1 ms, 100  μm lateral resolution would require an A-scan rate of at least 2.5 MHz. In theory, the elastogram lateral resolution is limited by the minimum distance at which a wave travels before being detected, and is related to the wavelength and speed of the traveling wave in addition to system phase sensitivity. In practice, lateral resolution is more appropriately determined by the frame rate. It follows that softer tissue samples, such as the lens, may be imaged with microscale resolution due to slower wave speeds. In dynamic OCE, it is clear that scan speed remains a limitation to high-resolution elastography and will continue to be an area of focus.

## 5 Discussion

### 5.1 Technical

Over the last decade, OCE has emerged as a viable tool to noninvasively probe the biomechanics of the eye, especially the anterior segment. A properly designed OCE system can complement other modalities, where differences in elasticity can potentially help identify tissue types related to disease and monitor therapeutic interventions. As noted in Fig. 1, an OCE system uses three steps to produce information related to tissue elasticity: (1) mechanical loading, (2) tissue response, and (3) motion detection. From maps of tissue displacements and strains detected with OCT, the goal of OCE is to produce images of tissue elastic and viscoelastic properties, and ultimately quantitative maps of the static (i.e., low-frequency) Young’s modulus.

Mechanical loading methods are static and dynamic (Fig. 2) using both contact and noncontact approaches. For clinical applications in the anterior segment of the eye, it appears that dynamic, noncontact methods are the most translatable. Current noncontact methods included air-puff excitation (Fig. 13), optical excitation (Fig. 14), and AμT using air-coupled ultrasound (Fig. 15). For measurements in the cornea (and sclera), optical excitation and AμT provide high bandwidth and spatially precise excitation well-matched to mechanical wave analysis. It is expected that as these technologies mature, they will be the preferred method for noncontact, dynamic loading.

Soft tissue responds to dynamic loading by launching mechanical waves (Fig. 16). Narrowband excitation can be used to map mechanical resonances related to both viscoelastic parameters and sample geometry. Broadband excitations produce mechanical waves, which in bulk materials exhibit wave speed and attenuation directly related to tissue viscoelastic parameters. For geometries close to those encountered in the anterior segment of the eye, surface and guided waves are produced with dispersion related to both viscoelastic and geometric parameters. However, high-frequency limits of wave propagation parameters in bounded media can produce estimates of tissue elasticity independent of sample geometry. This result suggests that robust, quantitative elasticity maps of structures within the anterior segment of the eye, especially the cornea, are possible.

Micro- and nanoscale tissue motion can be imaged using either speckle tracking of OCT scans, or differential phase processing of PhS-OCT scans, or some combination thereof. For large motion, speckle tracking has distinct advantages because it can measure displacements exceeding an optical wavelength and produce multidimensional displacement estimates. For smaller motion, however, PhS-OCT is much superior to speckle tracking since its ultimate precision is limited only by the SNR of the OCT signal. At typical SNRs for clinical imaging (between 20 and 50 dB), nanometer-scale displacements can be easily detected. Differential phase measurements in PhS-OCT obtained at very high frame rates match well to noncontact dynamic loading methods for OCE of the anterior segment of the eye. Current scan protocols to achieve sensitive phase measurement include M-B and B-M modes. While M-B provides exquisite phase sensitivity to small tissue motion, the total time required to acquire volumetric information of the sample is relatively long (>minutes), not optimal for clinical translation where imaging time is an important factor. B-M mode provides much faster imaging that can dramatically reduce the total time required for OCE (<1  s), but requires an ultrafast OCT system of MHz imaging speed.

Recent advances in MHz swept-laser sources can be leveraged to produce OCT frame rates (>kHz) fast enough to capture propagating mechanical waves within biological tissue.^58^^,^^137^ Further advances in SS-OCT systems suggest that multi-megahertz A-line rate systems can provide high-quality, and even phase-stable, OCT scans,^144^ leading to high-speed dynamic OCE with high-resolution multi-kilohertz imaging based on configurations similar to that of Fig. 17(b). Compared to slower SD-OCT systems, however, the phase noise in SS-OCT systems remains a major issue to overcome in the future development of clinically translatable systems. Instead of relying on a MHz swept-laser source, another practical future alternative to reduce total imaging time is to perform parallel imaging through multiple channels,^145^ with each channel targeted to different regions of the sample.

Multiple methods can image tissue viscoelasticity in the eye using the basic steps of mechanical loading, tissue response, and OCT-based motion detection. Based on the specific application, many of the ones presented in this review can provide qualitative information to help identify specific tissue types using elastic and viscoelastic properties. All, however, fall short of truly accurate quantitative maps of the static (i.e., low-frequency) Young’s modulus needed to drive biomechanical models predicting shape changes in the primary focusing apparatus of the eye. Static methods require simultaneous maps of both stress and strain to estimate the Young’s modulus. Dynamic methods are highly influenced by sample geometry. Even propagating, high-bandwidth mechanical waves are subject to both geometric (Fig. 8) and material (Fig. 10) dispersion that must be taken into account to produce quantitative maps of the Young’s modulus.

Recent attempts to quantify the elastic modulus based on computational methods accounting for specific corneal constraints include those using the Rayleigh–Lamb frequency equation (RLFE)^10^ and the finite element method (FEM).^146^ Analysis of the fluid–tissue interface taking into account complex corneal structure was experimentally shown to affect wave behavior independent of elastic modulus based on an FEM approach.^147^ A modified RLFE method has also been demonstrated that accounts for air/tissue and fluid/tissue interfaces.^9^ Other physical effects, such as corneal hydration,^147^ have been shown to complicate the behavior of propagating mechanical wave in tissue and must be accurately modeled. Together with continued development of optimal and clinically adaptable OCT systems to monitor tissue motion, future experiments and computational analysis focused on corneal-specific wave behavior will likely be an important step in transient OCE development.

**Fig. 24:**
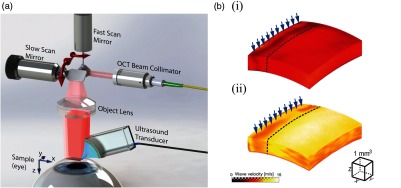
(a) Schematic demonstrating noncontact system using AμT to analyze corneal biomechanics. (b) 3-D renderings of group velocity within the cornea demonstrate different elasticity at different IOP—top 10 mm Hg and bottom 40 mm Hg. (Figure reproduced from Ref. 58.)

Although truly robust and accurate modulus quantitation has been difficult to demonstrate for the complex geometry of the anterior segment of the eye, recent studies with very broadband mechanical waves launched at the cornea–air interface suggest that reliable elastic modulus quantitation may be possible in the cornea. Using the AμT approach described in Refs. 57 and 58 and shown in Fig. 24(a), OCE measurements of the cornea were obtained in a pig model. 4-D images of cornea structure and mechanical wave propagation, such as the ones shown in Fig. 22, were recorded as the IOP was changed, and group velocity images derived from wavefield data [Fig. 24(b)] were used to qualitatively show changes in cornea elasticity at different IOP.

**Fig. 25:**
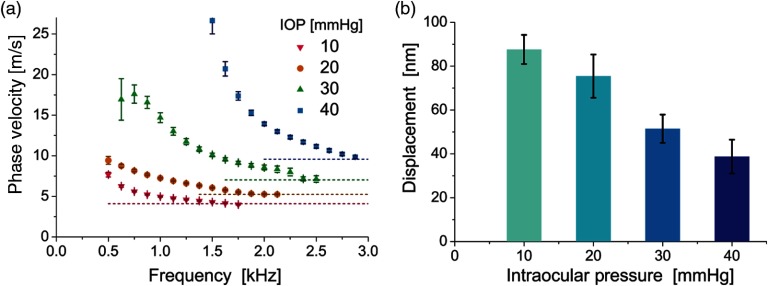
(a) Phase velocity as a function of frequency measured with OCE in a region of pig cornea for different values of the IOP. (b) Surface displacement of the cornea in the same cornea regions for a known mechanical force delivered with AμT at different IOP. (Figure reproduced from Ref. 58.)

Because of their wide bandwidth, these same data can be used for regional dispersion analysis to compare with expected curves of the type presented in Fig. 8. The result of this analysis for a small region of the cornea is presented in Fig. 25(a) where the phase velocity is plotted as a function of frequency over the experimental bandwidth at different values of the IOP. Clearly, a high-frequency asymptote of the type shown in Fig. 8 is apparent in these measurements. This asymptote can be used to compute the shear (Young’s) modulus in that region independent of the specific cornea geometry and relatively independent of frequency, assuming that material-dependent attenuation is small over the same frequency range. If material-dependent attenuation is significant (e.g., Fig. 17), then a complete mechanical model including both forms of dispersion must be developed for robust elasticity reconstruction. Nevertheless, these results suggest that robust regional dispersion analysis of broadband bounded waves can potentially yield quantitative maps of the Young’s modulus in the cornea that can drive biomechanical models predicting cornea shape changes due to changing IOP and potential therapeutic interventions.

In addition to quantitative Young’s modulus images, OCE can also provide information directly related to the IOP. Figure 25(b) shows that the amplitude of the mechanical displacement wave averaged within the excitation region decreases with IOP, consistent with previous US-based acoustic radiation force impulse imaging studies in many tissues showing a strong correlation between higher wave speeds and smaller displacements.^148^ Combining quantitative maps of the Young’s modulus determined from dispersion analysis [Fig. 25(a)] with simultaneous maps of corneal displacement for a known radiation force [Fig. 25(b)] and corneal thickness measurements obtained from OCT imaging can potentially drive biomechanical models to estimate IOP without any assumptions about cornea mechanical properties. The interrelationship among corneal thickness, corneal geometry, IOP, and tissue response has recently begun to be explored.^149^ This similar approach will be tested in future studies and quantitatively compared to current clinical IOP measurement devices that must assume some average elastic properties for the cornea.

Future technical studies will expand on recent developments, pushing toward higher quality OCE images and more reliable Young’s modulus maps. We expect that work will focus on four areas: better sources of noncontact dynamic loading, faster PhS-OCT systems, scan protocols with improved phase noise characteristics, and quantitative reconstruction algorithms leveraging broadband wavefield measurements to remove the effects of both geometric and material dispersion from estimates of the static Young’s modulus. Other noncontact methods will likely continue to be explored, including passive elastography that uses natural vibrations induced in the cornea by physiologic sources to estimate mechanical properties.^150^ Advancements in each of these areas will affect system design and influence choices made in other areas. For example, if phase noise can be dramatically reduced in SS-OCT systems providing the frame rates required for real-time 4-D data acquisition, then optical excitation methods may be preferred over air-coupled ultrasound because they are much easier to integrate into an all-optical OCE system. Clearly, with the rapid development of technologies relevant to OCE, we can expect continued advances in system performance over the next decade rivaling those made in the last decade.

### 5.2 Clinical

To this point, we have focused primarily on technical issues of probing mechanical properties with relatively noninvasive OCE methodologies. In this section, we explore potential applications where OCE may have significant impact on current clinical practice. We then discuss current challenges and future directions to highlight the path toward clinical translation of the OCE methods described in this review.

The human eye is a complex optic system at the beginning of the visual system designed to deliver an external image to the visual cortex in the central nervous system. This partially transparent, mechanically stable, optic system contains numerous structures, including the cornea, lens, sclera, retina, trabecular meshwork, and optic nerve. Fundamentally, ocular structures are interconnected to focus light passing through the cornea and lens onto the retina where optical signals are transduced. The eye’s mechanical structure plays an important role in physically focusing light as well as indirectly regulating much of its function. The human eye includes an outer layer of collagen tissues, referred to as the cornea and sclera, forming an envelope around the globe that connects at the limbus. The outer corneoscleral envelope protects the inner portion and is often the subject of mechanical probing. The epithelium and endothelium are not directly responsible for mechanical properties, but they do play a role in regulating corneal hydration which is known to alter mechanical properties to some degree.^147^

Interest in corneal biomechanics has grown greatly, especially with the advent of the ocular response analyzer (ORA, Reichert Technologies) in 2005 and dynamic Scheimpflug analyzer (DSA, Corvis ST, Oculus Opitkgerate GmbH) in 2012. Over 100 papers are now published annually on the topic, and the *Journal of Cataract and Refractive Surgery* devoted an entire recent issue to the subject.^151^ These tools could potentially influence clinical decisions ranging from surgical interventions to monitoring procedures such as collagen crosslinking therapies. Nevertheless, they have significant limitations.^152^ In particular, they provide average viscoelastic properties of the entire cornea that greatly depend on the experimental conditions rather than detailed spatial maps of fundamental material parameters required for robust biomechanical models. Also, they do not account for the highly nonlinear stress–strain relation, another important component of a robust biomechanical model.

Precise knowledge of corneal biomechanics is critical for early diagnosis, optimal management of diseased corneas (e.g., keratoconus) and predicting the risks of surgical intervention of healthy corneas, such as post-LASIK ectasia. In addition, traditional IOP measurements using direct contact are often confounded by the elastic properties of the cornea.^152^ Instruments such as the ORA and DSA attempt to account for these properties by monitoring the corneal response to a dynamic mechanical stimulus as part of an IOP measurement. Dynamic tonometry is being considered as a potential screening tool for glaucoma and myopia, where there is recent evidence that corneal elasticity is linked to disease progression for these conditions affecting 70% of the population.^153^ However, it is clear that these measurements are highly susceptible to experimental conditions and cannot be used to map fundamental corneal viscoelastic parameters at high spatial resolution.^152^

Currently, topography, pachymetry, tonometry, and to a lesser extent OCT are the primary clinical tools to probe corneal biomechanics. Diseased as well as healthy corneas can benefit from better understanding and measurement of biomechanics. For example, Keratoconus, a noninflammatory corneal degeneration characterized by progressive weakening with protrusion and thinning of the cornea (occurs 1 in 2000 of the population), is often found with rapid progression in young adults (from late teens to mid-twenties) and is often missed for high astigmatism or myopia until vision is uncorrectable.^154^^–^^156^ Curvature, elevation, and pachymetric changes are secondary signs of keratoconus, so early detection enabling effective treatment is not possible with current screening tools and may only be possible with a personalized biomechanical model based on spatial maps of fundamental material properties. Reduced corneal stability has also been reported in diseases such as Trisomy 21 (Down Syndrome), Ehler–Danlos syndrome, and osteogenesis imperfecta.^4^ Therapeutic interventions, such as UV crosslinking, have been monitored with OCE to measure the progression and retardation of collagen degradation.^9^^,^^75^^,^^136^ Comparable OCE results have been reported on rabbit corneas using formalin crosslinking^56^ and rose-bengal/green light crosslinking.^146^ However, currently, there are no methods available to quantify baseline corneal biomechanics of diseased corneas with the spatial resolution and accuracy required to robustly predict treatment outcomes, potential complications, and long-term stability. There is clearly a long-term clinical opportunity for high-resolution OCE systems.

Other clinical procedures may also benefit greatly from high-resolution maps of corneal elasticity. Currently, topography and pachymetry are the primary screening tools to evaluate healthy corneas for refractive surgery, an iatrogenic corneal weakening procedure to correct refractive errors. The selection criteria for LASIK and photorefractive keratectomy are based on empirically established parameters; this procedure is now reserved for patients with corneal thickness more than 500  μm and normal topographic shape.^157^ Even for those who passed current screening criteria, more than 1% of LASIK patients will experience ectasia.^158^^–^^161^ This adds up to over 10,000 patients per year with a severe medical problem created by an elective procedure, even based on conservative selection criteria. The recent FDA PROWL study^162^ finds high rates of symptoms such as dry eye and visual symptoms such as halos after LASIK during the immediate postoperative period. Other studies report that additional long-term dissatisfaction of LASIK patients (50,000) were due to refractive regression and residual refractive error.^163^^,^^164^ Current refractive surgery planning uses a population-based average of corneal biomechanics rather than an individualized treatment plan, which produces individual variation in treatment outcomes even with the most conservative selection criteria. The availability of an accurate, personalized corneal biomechanical map of individual cornea from high-resolution OCE may enable a customized treatment plan for each patient, with the biomechanical response adequately predicted for the long term.

The mechanical properties of the human eye are thought to play an important role in the regulation of the IOP. This interrelationship is captured by the near-incompressible constitutive equation for soft tissue [Eq. (7)], where both modulus-dependent strain terms and the hydrostatic internal pressure contribute to the total internal stress. In addition, the elastic modulus of the cornea, and presumably other ocular tissues, is a function of the IOP.^9^^,^^58^^,^^136^^,^^149^^,^^165^ As the human eye retains its shape in part by regulating the IOP, the IOP may be a valuable clinical parameter to help diagnose and manage disease such as glaucoma.

Current clinical IOP measurements rely on indirect techniques with limited accuracy because ocular biomechanics cannot be taken into account on a patient-by-patient basis.^166^^,^^167^ Available systems used to measure IOP *in vivo*, such as Goldmann applanation, ORA, and dynamic contour and noncontact tonometry, may be improved by calibration methods that account for corneal mechanical properties. Additionally, it is difficult for conventional (not OCE based) tonometry systems to directly visualize the distribution of local corneal mechanical properties in addition to providing robust estimates of quantitative modulus values. As current clinical gold standards struggle to accurately estimate the influence of corneal mechanical properties on IOP, approaches utilizing available systems may lead to misinformed clinical decision making, a niche where OCE may find great utility (as suggested in the previous section). Recent advances in OCT have not only made OCE possible for IOP-related application and mapping of corneal biomechanical parameters but also have led to system developments that show great clinical promise for additional ophthalmic applications.

For example, OCE has also shown utility in lens analysis. The lens focuses light onto the retina, and it is dynamically shaped by ciliary muscles through its elastic response to intrinsically induced stress. With aging, it becomes less pliable and loses the ability to accommodate (the process of changing focus from near to far).^168^ This loss is associated with reduced accommodation and may be directly measurable using OCE methods, as demonstrated using ARF-OCE to detect age-related stiffness in rabbit lens.^76^ It has, however, also been reported that increased IOP affects the shear wave speed in the lens,^169^ implying a less understood IOP dependence on lens mechanical moduli.

Behind the lens, the vitreous body transmits pressure from the anterior chamber to the interior portion of the eye, placing a preload on retinal tissue. Elevated IOP may thus play a role in damaging the posterior segment under increased pressure. ARF-OCE has also been demonstrated as a mean to assess elasticity within the retina,^18^ potentially providing additional information regarding cellular degradation from measurements of retinal and choroidal stiffness, as well as providing further insight into how IOP affects ocular function.

From a mechanical viewpoint, OCE can probe different features that may potentially provide clinicians with additional valuable information regarding pathology. For example, ocular tissues have been reported to have a range of moduli, nonlinearities, and apparent anisotropy that may be inherently linked to tissue structure and function. However, there remain a number of challenges in detecting such biomechanical features that must be addressed.

First, the cornea, and most ocular tissue types for that matter, are not homogeneous, but are layered with mechanical properties that vary by position and depth. The structure of ocular tissues often produces complex shapes making it difficult to quantify stress distributions and wave behavior. The structure and shape are further complicated by changes in IOP, which alter both thickness and curvature.

Different tissue types are also bounded by different media. For example, corneal tissue is bounded by air on one side and fluid on the other. The fluid interface has been shown to affect tissue thickness through hydration and must be accounted for. In complex structures such as the cornea, wave disturbances are guided and often include a number of propagating modes. In transient OCE, these modes must be considered for proper assessment of viscoelastic properties based on phase velocity and attenuation measurements. Accurate and quantitative viscoelastic assessment based on mechanical wave propagation needs continued work to appropriately decouple wave behavior based on mechanical properties from experimental conditions such as thickness, geometric constraints, and corneal hydration. As noted in the previous section, computational algorithms will likely play an important role in applying an appropriate mechanical model to describe wave propagation.

Once reliable and robust measurements of elastic modulus become possible in a noninvasive manner, the micro- and macrostructure of tissue may be used to infer vast amounts of pathophysiological data. Different ocular tissue types have unique structures that each serves a specific role. Each structure may serve an independent purpose, but is clearly interconnected with surrounding tissue to create an intricate system providing both structure and function to the optic system. Translating biomechanical assessment of the anterior segment into clinical practice may provide clinicians with important information on the specific role of unique ocular tissue types, but will likely require a much better understanding and characterization of the critical load-bearing tissues, such as the sclera. Final clinical applications might also require measurements of multiple OCE derived parameters such as local modulus and IOP.

Because imaging of elastic properties has not been possible *in vivo* previously, there exists a vast space for exploration and application. For OCE to truly have clinical impact, academic researchers, ophthalmologists, clinician-researchers, and engineers must work together to foster an environment that can recognize, detect, and address the influence of biomechanical forces on ocular tissues. Through such collaboration, OCE will continue to grow, and may very soon be applied in ophthalmology to map the biomechanics of the human eye at higher resolution and sensitivity than previously possible.
